# Circular RNA hsa_circ_101555 promotes hepatocellular carcinoma cell proliferation and migration by sponging miR-145-5p and regulating CDCA3 expression

**DOI:** 10.1038/s41419-021-03626-7

**Published:** 2021-04-06

**Authors:** Xiaoguang Gu, Jianan Zhang, Yajuan Ran, Hena Pan, JinHong Jia, Ying Zhao, Xijuan Zhao, Wendi Li, Shasha Song, Xiufeng Yu

**Affiliations:** 1 College of Medical Laboratory Science and Technology, Harbin Medical University (Daqing), 163319 Daqing, P. R. China; 2 Central Laboratory of Harbin Medical University (Daqing), 163319 Daqing, P. R. China; 3 Department of Pharmacy, The Second Affiliated Hospital of Chongqing Medical University, 400010 Chongqing, P. R. China; 4 Department of Pharmacology, Harbin university of commerce, 150081 Harbin, P. R. China; 5 College of Pharmacy, Shenzhen Technology University, 518118 Shenzhen, P.R. China

**Keywords:** Liver cancer, Long non-coding RNAs

## Abstract

Circular RNAs have been reported to play significant roles in regulating pathophysiological processes while also guiding clinical diagnosis and treatment of hepatocellular carcinoma (HCC). However, only a few circRNAs have been identified thus far. Herein, we investigated the role of a specific closed-loop structure of hsa_circ_101555 that was generated by back-splicing of the host gene casein kinase 1 gamma 1 (*CSNK1G1*) in the development and proliferation of HCC. We investigated the expression of Hsa_circ_101555 in HCC and normal tissues using bioinformatics. The expression level of hsa_circ_101555 was further detected by fluorescence in situ hybridization and qRT-PCR in ten HCC patients. Transwell, migration, WST-1 assays, and colony formation assays were used to evaluate the role of hsa_circ_101555 in HCC development and proliferation. The regulatory mechanisms of hsa_circ_101555 in miR-145-5p and CDCA3 were determined by dual luciferase reporter assay. A mouse xenograft model was also used to determine the effect of hsa_circ_101555 on HCC growth in vivo. hsa_circ_101555 showed greater stability than the linear RNA; while in vitro and in vivo results demonstrated that hsa_circ_101555 silencing significantly suppressed cell proliferation, migration, and invasion of HCC cells. Rescue experiments further demonstrated that suppression of miR-145-5p significantly attenuated the biological effects of hsa_circ_101555 knockdown in HCC cells. We also identified a putative oncogene *CDCA3* as a potential miR-145-5p target. Thus, our results demonstrated that hsa_circ_101555 might function as a competing endogenous RNA of miR-145-5p to upregulate CDCA3 expression in HCC. These findings suggest that hsa_circ_101555 may be a potential therapeutic target for patients with HCC.

## Introduction

Hepatocellular carcinoma (HCC), the most prevalent form of primary liver cancer, represents one of the most common malignant tumors globally^1^. HCC is an aggressive disease with dismal prognosis, constituting the third leading cause of cancer-related deaths worldwide^2^. Despite advances in the clinical understanding of the underlying mechanisms in HCC development and progression, its 5-year survival rate remains low^3^. Additionally, the molecular pathogenesis and therapeutic targets in HCC remain largely unknown. Therefore, understanding the pathogenic process of HCC and its regulatory mechanisms would significantly aid its management.

Circular RNAs (circRNAs) are a newly discovered noncoding RNA (ncRNA) that ubiquitously exist in several species^4,5^. Unlike canonical linear RNAs, circRNAs form a covalently closed continuous loop structure that lack 5′ caps and 3′ polyadenylated tails, making them more stable than linear RNAs^6^. circRNAs reportedly function as molecular sponges for microRNAs (miRNAs)^7,8^ and RNA-binding proteins^9^, while also serving as vital regulators of gene transcription and expression^10,11^. Furthermore, circRNAs are highly conserved across multiple species and exhibit tissue-specific and development stage-dependent expression patterns^12,13^. These features imply that circRNAs possess significant functions in biological and pathological processes.

Recent studies have reported that circRNAs are differentially expressed in HCC and serve a central role in its carcinogenesis and progression^14–20^. Thus, circRNAs may represent promising diagnostic markers and therapeutic targets for HCC. However, compared with other ncRNAs, such as miRNAs and long noncoding RNAs, the research on circRNA in HCC remains in its infancy. To date, only a few functional circRNAs have been discovered and characterized in HCC^21^, while a large number remain to be explored or identified.

In the present study, we analyzed the expression profiles of three circRNA in human HCC tissues and identified hsa_circ_101555 to be conserved and significantly upregulated. Therefore, we focused on investigating the role of hsa_circ_101555 in the development and proliferation of HCC with respect to the miR-145-5p/CDCA3 signaling axis.

## Results

### hsa_circ_101555 is upregulated in HCC

**Fig. 1:**
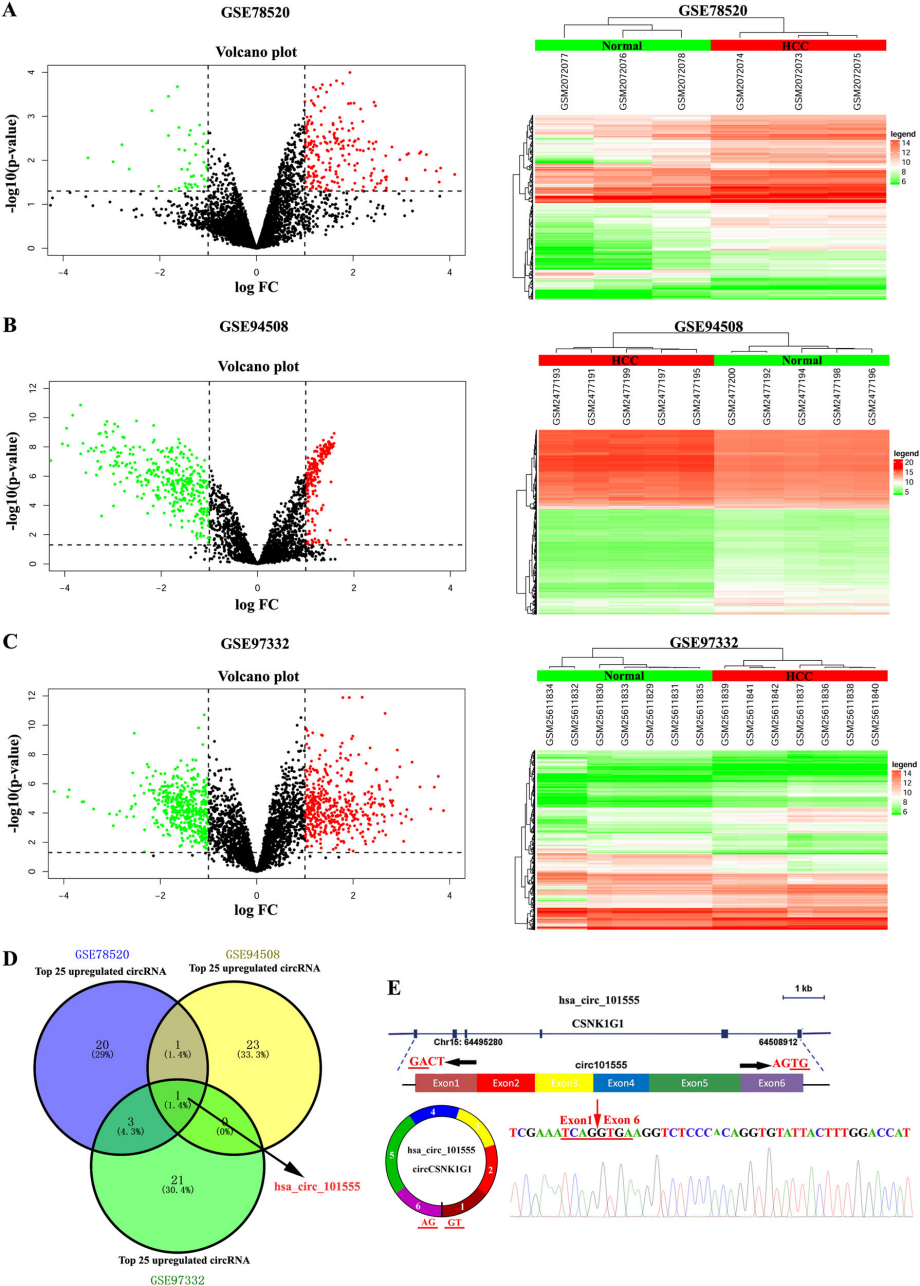
circRNA expression profiles in HCC and paired nontumorous samples. **A**–**C** Volcano and scatter plots show variation in circRNA expression between HCC and nontumor liver tissues from the GSE7852 (**A**), GSE94508 (**B**), and GSE97322 (**C**) datasets. **D** Venn diagram. **E** hsa_circ_101555 consists of six exons (exon 1–6, 815 nt) and is derived from the host gene *CSNK1G1*. The existence of the back-splice junction was confirmed by Sanger sequencing.

To identify unique circRNAs involved in HCC, we analyzed the microarray data of GSE7852, GSE94508, and GSE97322 datasets downloaded from the Gene Expression Omnibus (GEO) database. We then visualized the differentially expressed circRNAs (DEcircRNAs) in HCC and normal tissue samples using the “limma” package of R software. A false discovery rate < 0.05 and |log2fold-change > 1 were set as the cutoff criteria for screening the DEcircRNAs (Fig. 1A–C). Among the top 25 upregulated DEcircRNAs, only hsa_circ_101555 appeared in all three GSE datasets (Fig. 1D). Thus, we focused on investigating its role in HCC progression. Subsequently, according to circBase (http://www.circbase.org), we found that hsa_circ101555 was derived from the host gene casein kinase 1 gamma 1 (*CSNK1G1*), consisting of six exons (exon 1–6, 815 nt) cyclized by the head-to-tail splicing of exon 1 and exon 6. The existence of a back-spliced junction was confirmed by Sanger sequencing (Fig. 1E). We further verified the expression of *CSNK1G1* in HCC tissues, and found it to be upregulated (Supplementary Fig. 1A). Moreover, analysis of the overall survival and *CSNK1G1* expression using GEPIA (http://gepia.cancer-pku.cn/) revealed that high *CSNK1G1* expression in HCC was not associated with overall survival (Supplementary Fig. 1B-C).

### hsa_circ_101555 is primarily localized in HCC cell cytoplasm

**Fig. 2:**
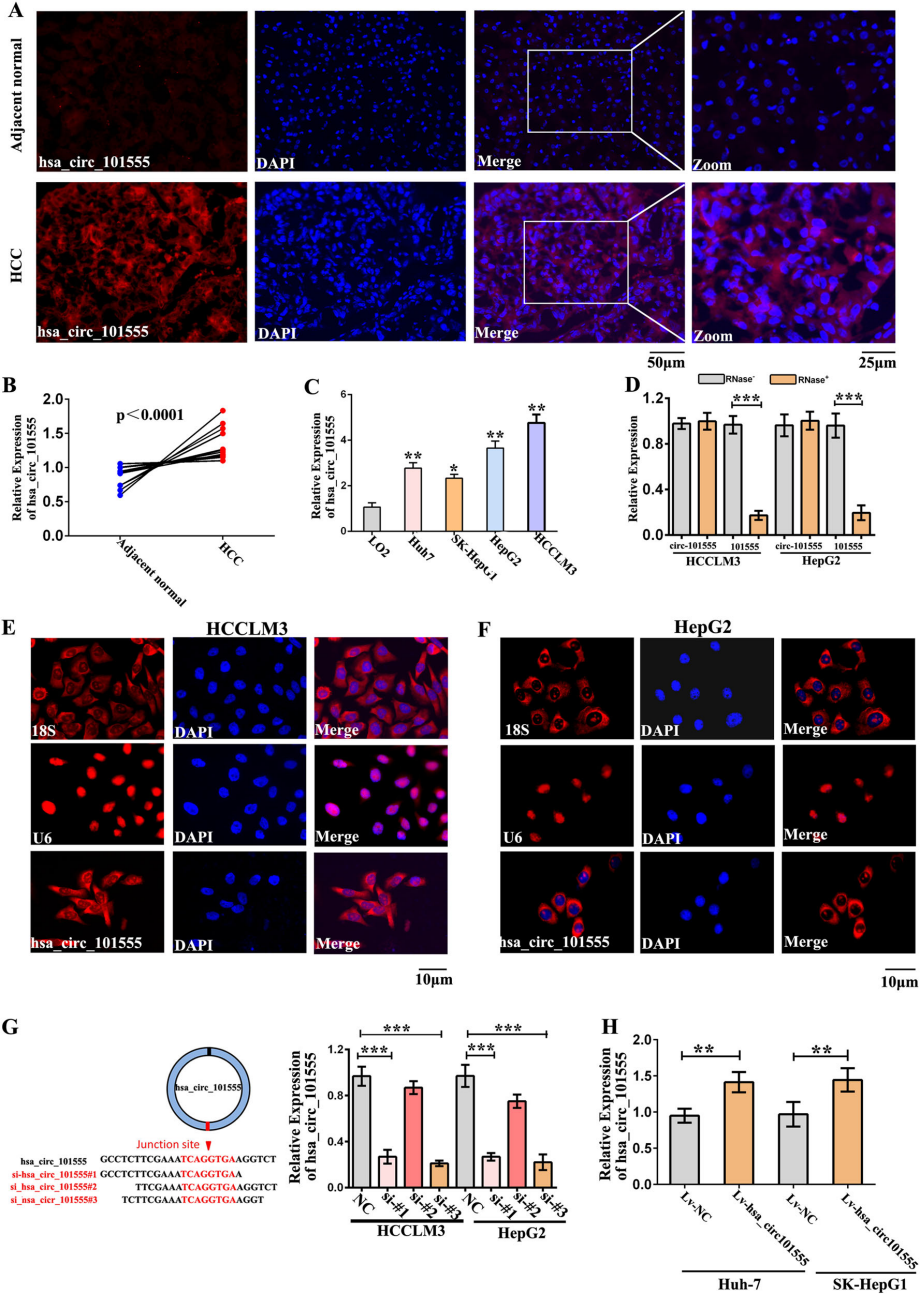
hsa_circ_101555 localizes in cytoplasm. **A** hsa_circ_101555 in HCC tumor and adjacent nontumor tissues detected by FISH. **B** Expression of hsa_circ_101555 in ten HCC tumor and adjacent normal tissues measured by qRT-PCR (***p < 0.001). **C** Expression of hsa_circ_101555 in multiple HCC cell lines (*P < 0.05; **P < 0.01; *n* = 4). **D** Linear 101555 is readily digested by RNase R, while hsa_circ_101555 is not (***P < 0.001; *n* = 4). **E**, **F** FISH confirmed that hsa_circ_101555 is predominantly located in cytoplasm. Nuclei stained with DAPI. U6, 18 S, and hsa_circ_101555 labeled with cy3 (*n* = 4). **G** Diagrammatic representation of siRNA targeting the back-splice junction (si-hsa_circ_101555-#1, si-hsa_circ_101555-#2, and si-hsa_circ_101555-#3); qRT-PCR results for hsa_circ_101555 expression in HCCLM3 and HepG2 cells treated, with or without siRNA. si-NC, control oligonucleotides with scramble sequence; si-hsa_circ_101555-#1, si-hsa_circ_101555-#2, and si-hsa_circ_101555-#3, oligonucleotides targeting the back-splice junction (***P < 0.001; *n* = 4). **H** qRT-PCR results for hsa_circ_101555 in Huh-7 and SK-Hep-1 cells treated with lv-NC or lv- hsa_circ_101555 (**P < 0.01; *n* = 4).

In general, the subcellular localization of circRNA determines its primary mode of action. Fluorescent in situ hybridization (FISH) analysis revealed that hsa_circ_101555 expression was higher in tumor tissues than in matched nontumor sections (Fig. 2A). Furthermore, qRT-PCR examination confirmed that hsa_circ_101555 expression was markedly higher in HCC tissues than in adjacent normal tissues (Fig. 2B). High hsa_circ_101555 expression in HCC was associated with overall survival (Supplementary Fig. 2A).

We also analyzed hsa_circ_101555 expression within the serum of HCC patients and healthy controls and found the expression to be significantly higher in HCC patients compared to healthy controls (Supplementary Fig. 3A). Meanwhile, we also observed that hsa_circ_101555 was upregulated in III–IV stage HCC compared to I–II stage patients, based on TNM stage (Supplementary Fig. 3B). Additionally, the expression of hsa_circ_101555 was higher in all HCC cell lines (HepG2, SK-Hep-1, Huh-7, and HCCLM3) compared to normal liver LO2 cells, with a significantly higher expression in HepG2 and HCCLM3 cells than in Huh-7 and SK-Hep-1 cells (Fig. 2C).

The circular structure of hsa_circ_101555 was confirmed using RNase R. As shown in Fig. 2D, amplified linear transcripts of CSNK1G1 were clearly degraded by RNase R, while hsa_circ_101555 was resistant to degradation. These data demonstrated both the presence and circular structure of hsa_circ_101555 in HCC cells (HepG2 and HCCLM3). Moreover, hsa_circ_101555 was predominantly located in the cytoplasm (Fig. 2E–F).

### hsa_circ_101555 acts as an oncogene in HCC

To further investigate the regulatory role of hsa_circ_101555, we designed three hsa_circ_101555 small interfering RNAs (siRNAs) to specifically target different binding sites on the back-splice junction sequence of hsa_circ_101555. As si-hsa_circ_101555_001 and si-hsa_circ_101555_003 effectively silenced hsa_circ_101555 expression in HCCLM3 and HepG2 cell lines, they were used for subsequent experiments (Fig. 2G). We also generated stable hsa_circ_101555 overexpressing Huh-7 and SK-Hep-1 cells through lentiviral transduction and observed that lv-hsa_circ_101555 treatment caused marked upregulation of hsa_circ_101555 in these cells (Fig. 2H).

**Fig. 3:**
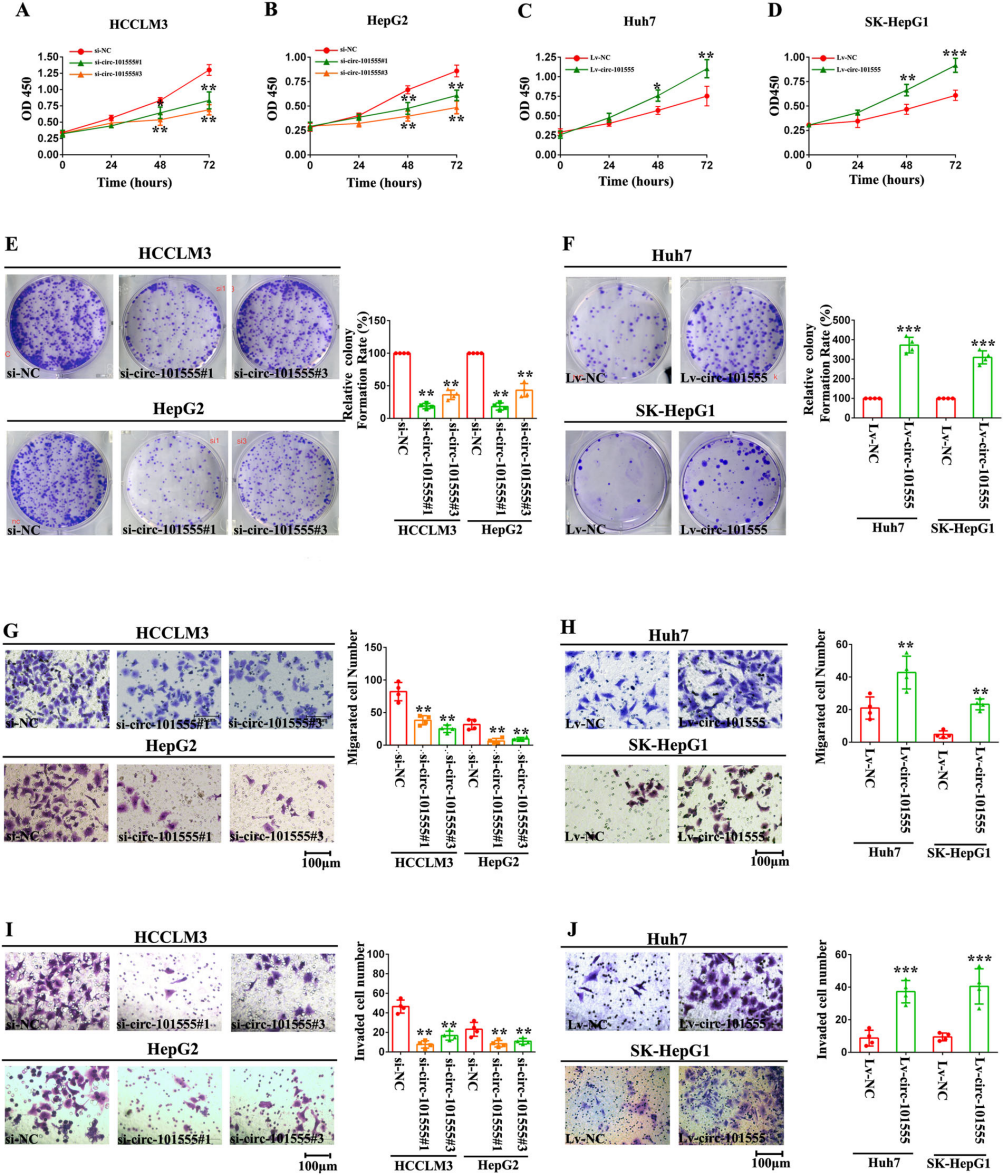
hsa_circ_101555 acts as an oncogene in HCC. **A**, **B** Proliferative capacity of HCCLM3 and HepG2 cells transfected with siRNA-hsa_circ_101555, or siRNA-NC, evaluated by WST-1 assay (*P < 0.05; **P < 0.01; *n* = 4). **C**, **D** WST-1 assay results in lv- hsa_circ_101555 transfected Huh-7 and SK-Hep-1 cells (*P < 0.05; **P < 0.01; ***P < 0.001; *n* = 4). **E** Colony formation assay evaluating the proliferative ability of HCCLM3 and HepG2 cells transfected with siRNA-hsa_circ_101555 or siRNA-NC (**P < 0.01; *n* = 4). **F** Effect of hsa_circ_101555 overexpression on Huh-7 and SK-Hep-1 cells examined by colony formation assay (***P < 0.001; *n* = 4). **G** Cell migration capability of HCCLM3 and HepG2 cells transfected with siRNA-hsa_circ_101555 or siRNA-NC, assessed by transwell migration (**P < 0.01; *n* = 4). **H** Effect of hsa_circ_101555 overexpression on Huh-7 and SK-Hep-1 cells, examined by transwell migration (**P < 0.01; *n* = 4). **I** Cell invasion capacity of cells transfected with siRNA-hsa_circ_101555 or siRNA-NC, evaluated by Matrigel invasion assays (**P < 0.01; *n* = 4). **J** Effect of hsa_circ_101555 overexpression on Huh-7 and SK-Hep-1 cells, examined by Matrigel invasion assays (***P < 0.001; *n* = 4).

Subsequently, we detected the effect of hsa_circ_101555 knockdown and overexpression on HCC tumor progression in vitro. WST-1 assay results showed that hsa_circ_101555 silencing reduced HCCLM3 and HepG2 cell proliferation (Fig. 3A and B), whereas its overexpression had the opposite effect in these cells (Fig. 3C and D). Colony formation assays further revealed that hsa_circ_101555 was positively associated with HCCLM3 and HepG2 cell proliferation (Fig. 3E), whereas hsa_circ_101555 overexpression had the opposite effect on Huh-7 and SK-Hep-1 cells (Fig. 3F). Notably, proliferating cell nuclear antigen levels in these cells were initially elevated, however, became attenuated following transfection with the hsa_circ_101555 back-splice junction-specific siRNA (Supplementary Fig. 4A–B).

HCCLM3 and HepG2 cell migration and invasion were also suppressed by hsa_circ_101555 silencing (Fig. 3G and I), whereas these properties were enhanced by hsa_circ_101555 overexpression in Huh-7 and SK-Hep1 cells (Fig. 3H and J). We further evaluated the effect that hsa_circ_101555 has on the expression of migration markers in HCC cell lines. Western blot analysis revealed that hsa_circ_101555 silencing inhibited the expression of fibronectin and vimentin, while that of E-cadherin was increased in HCCLM3 and HepG2 cells (Supplementary Fig. 5A-B). Moreover, Hsa_circ_101555 overexpression significantly decreased the relative expression of E-cadherin, while increasing that of vimentin and fibronectin in both Huh-7 and SK-HepG1 cells (Supplementary Fig. 5C-D). Taken together, these results suggest that hsa_circ_101555 was required to sustain the proliferation, migration, and invasion of HCC cells in vitro.

### The RNA-binding protein EIF4A3 regulates hsa_circ_101555 expression

**Fig. 4:**
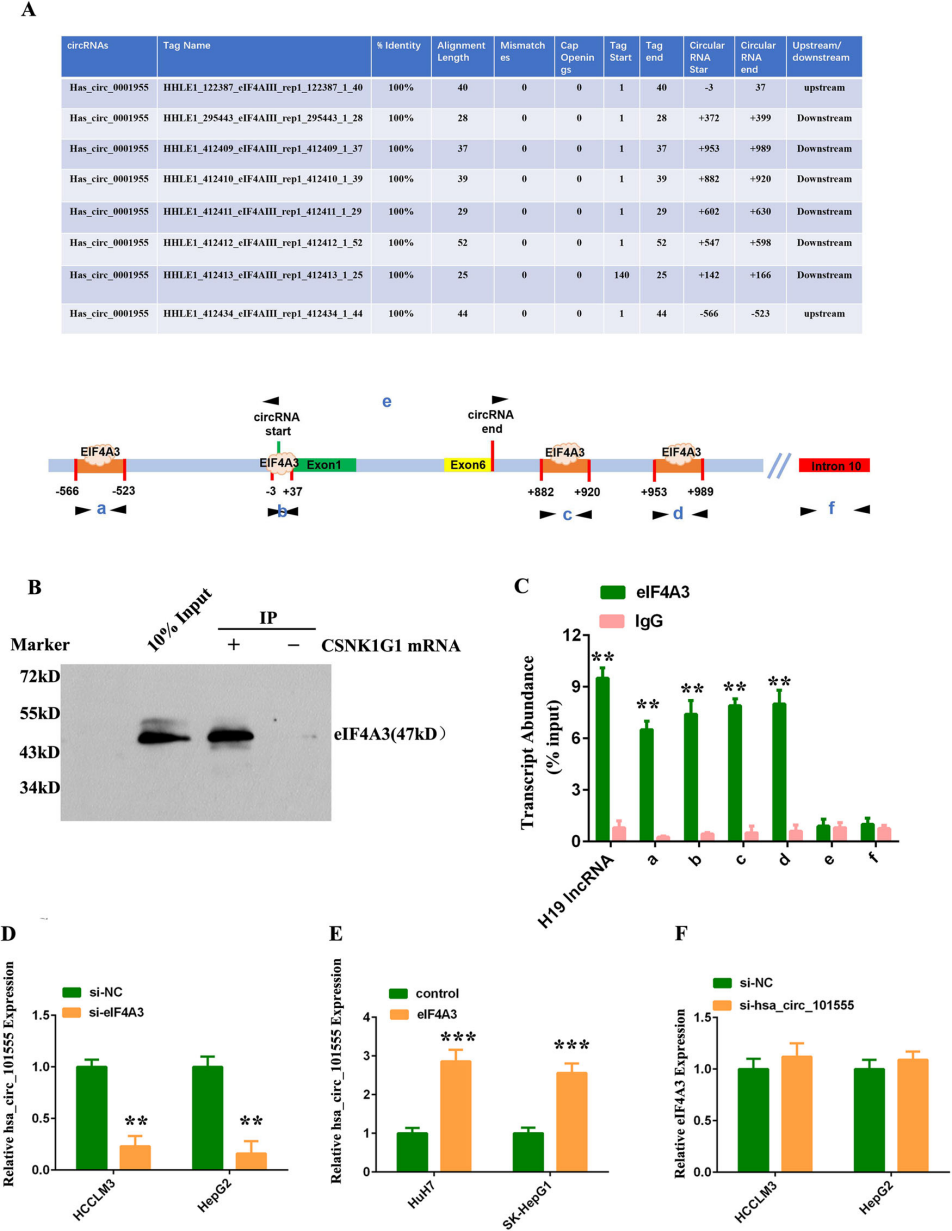
The RNA-binding protein EIF4A3 regulates hsa_circ_101555 expression. **A** Binding sites for EIF4A3 on the CSNK1G1 mRNA transcript, upstream or downstream of hsa_circ_101555, obtained from Circular RNA Interactome. **B** Pull-down assay confirms the enrichment of EIF4A3 protein in CSNK1G1 mRNA pull-down. **C** Quantitative real-time PCR following EIF4A3-RIP assay confirms the binding of EIF4A3 on the predicted binding region (a, b, c, and d) of CSNK1G1 mRNA. H19, a known interacting long noncoding RNA (lncRNA) with EIF4A3, served as the positive control. The intron 10 of CSNK1G1 mRNA was used as a negative control. **D** HCCLM3 and HepG2 cells transfected with control or siRNA targeted eIF4A3; hsa_circ_101555 expression detected by qRT-PCR. **E** HCCLM3 and HepG2 cells transfected with control or eIF4A3 overexpression plasmids; hsa_circ_101555 expression measured by qRT-PCR. **F** HCCLM3 and HepG2 cells transfected with control or siRNA targeted hsa_circ_101555; eIF4A3 expression detected by qRT-PCR.

EIF4A3, a core component of the exon junction complex, has been shown to play an essential role in pre-mRNA splicing^22^, while also inducing circRNA expression via binding to the upstream or downstream regions of host gene mRNA and inducing circular RNA formation^23–25^. In fact, we discovered four putative binding sites for EIF4A3 in the upstream and downstream region of the *CSNK1G1* mRNA transcript (CSNK1G1 pre-mRNA) via CircInteractome (https://circinteractome.nia.nih.gov/index.html) (Fig. 4A).

We conduced pull-down assay using CSNK1G1 mRNA and confirmed the enrichment of EIF4A3 protein in the pull-down of CSNK1G1 mRNA rather than control (Fig. 4B). The data from an RIP (RNA-binding protein immunoprecipitation) assay using anti-eIF4A3 antibody further indicated that eIF4A3 can bind CSNK1G1 mRNA through these four putative binding sites, which we designated as, a, b, c, and d, however, did not bind hsa_circ_101555 (designated e) in the corresponding RNA-protein complex (Fig. 4C). We then knocked down *eIF4A3* and observed reduced hsa_circ_101555 expression and increased *CSNK1G1* expression (Fig. 4C and Supplementary Fig. 6A). Meanwhile, overexpression of eIF4A3 increased hsa_circ_101555 and decreased host gene *CSNK1G1* expression (Fig. 4D and Supplementary Fig. 6B). In contrast, silencing hsa_circ_101555 did not affect EIF4A3 expression (Fig. 4E). Taken together, EIF4A3 was observed to increase hsa_circ_101555 expression, potentially by combining with the flanking sequences.

### Identification of miRNAs that binding hsa_circ_101555

**Fig. 5:**
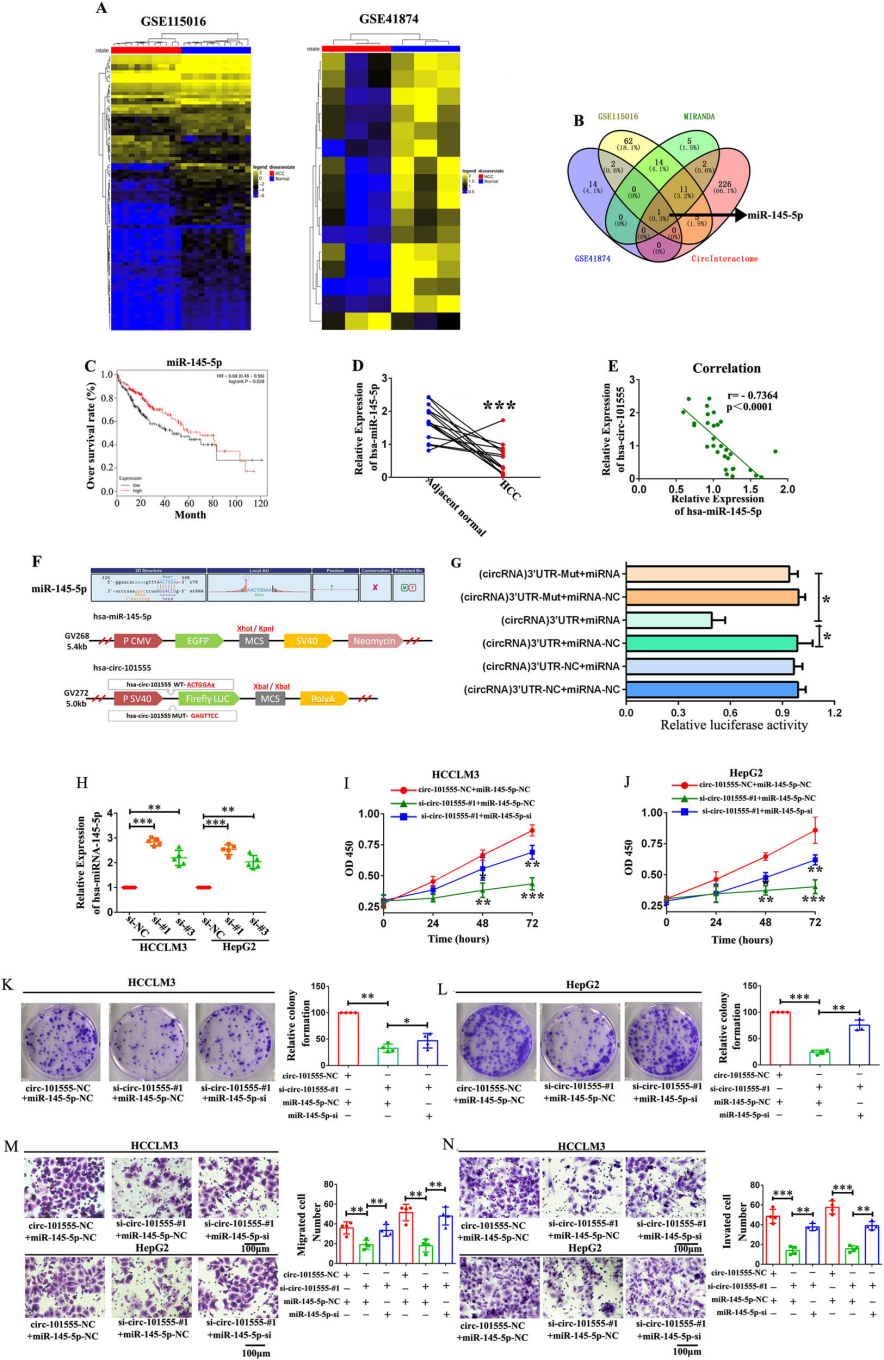
Identification of miRNAs binding to hsa_circ_101555 that modulate the proliferation, cell cycle, migration, and invasion of HCC cells via miR-145-5p. **A** Heatmap analyses exhibit differentially expressed miRNAs in HCC with |log2fold-change | > 1.0 and *P* < 0.05. **B** Venn analysis between predicted miRNA targeted by hsa_circ_101555 and differentially expressed miRNAs implying that miR-145-5p is involved in HCC. **C** Kaplan–Meier curves imply that patients with low miR-145-5p levels have a poorer 10-year overall survival. **D** Expression of miR-145-5p in 10 HCC tumor tissues and adjacent normal tissues, measured by qRT-PCR (***P < 0.001). **E** Pearson correlation analyses showing a correlation between hsa_circ_101555 and miR-145-5p expression (*n* = 20). **F**, **G** Schematic of hsa_circ_101555 wild-type (wt) and mutant (mut) luciferase reporter vectors. Luciferase reporter assay in HEK293T cells co-transfected with miRNA mimics, hsa_circ_101555-wt and mutant (mut) luciferase reporter vectors. **H** Expression of miR-145-5p analyzed by qRT-PCR in HCCLM3 and HepG2 cells transfected with siRNA-hsa_circ_101555-#1, siRNA-hsa_circ_101555-#3, or siRNA-NC (**P < 0.01; ***P < 0.001; *n* = 5). **I**, **J** Viability of HCCLM3 and HepG2 cells after co-transfection with siRNA-hsa_circ_101555 and miR-145-5p inhibitor measured using WST-1 assays (*P < 0.05;**P < 0.01; ***P < 0.001; *n* = 4). **K**, **L** Cell proliferation ability of HCCLM3 and HepG2 cells co-transfected with siRNA-hsa_circ_101555 and miR-145-5p inhibitor evaluated by colony formation assay (*P < 0.05;**P < 0.01; ***P < 0.001; *n* = 4). **M**, **N** Cell migration or invasion assays performed in HCCLM3 and HepG2 cells co-transfected with siRNA-hsa_circ_101555 and miR-145-5p inhibitor using transwell chamber, with or without Matrigel, respectively (**P < 0.01; ***P < 0.001; *n* = 4).

Given that circRNAs may act as competing endogenous RNAs (ceRNA) for miRNAs and regulate mRNA expression, we assessed the potential hsa_circ_101555 targets via a ceRNA-dependent mechanism. First, we determined the expression profiles of miRNAs from GSE115016 and GSE4187 datasets in HCC and normal tissue samples using miRNA microarray (Fig. 5A). Based on the target prediction tool, miRNAs were found to contain binding sites for hsa_circ_101555. Subsequent Venn analysis indicated the possible involvement of miR-145-5p in HCC (Fig. 5B). Therefore, we analyzed its expression in ten paired HCC and adjacent normal tissues by qRT-PCR and found it to be significantly downregulated in HCC tissues. Moreover, patients with low miR-145-5p level had a poorer 10-year overall survival (OV), compared to those with high miR-145-5p levels (Fig. 5C and D). Pearson correlation analysis further revealed a negative correlation (*p* < 0.0001 r = −0.7364) between hsa_circ_101555 and miR-145-5p expression levels (Fig. 5E). Additionally, luciferase reporter assays using either a wild-type hsa_circ_101555 sequence or a mutated miR-145-5p binding sites, inserted into the 3′ untranslated region of Renilla luciferase, showed that miR-145-5p overexpression significantly reduced the luciferase activity of the wild-type reporter without impacting that the mutant sequence-bound reporter (Fig. 5F–G). These findings indicate that hsa_circ_101555 may act as a sponge for miR-145-5p.

### hsa_circ_101555 modulates HCC cell proliferation, migration, and invasion via miR-145-5p

We further investigated whether hsa_circ_101555 affects the function of HCC cells via miR-145-5p by determining its expression levels in vitro. qRT-PCR revealed that hsa_circ_101555 silencing increased miR-145-5p levels in HepG2 and HCCLM3 cells (Fig. 5H). We also found that the proliferation (via WST-1 assay) and colony-forming ability of HCC cells co-transfected with si-hsa_circ_101555_003 and miR-145-5p inhibitor were higher than those of HCC cells transfected with only siRNA-hsa_circ_101555, suggesting that the downregulation of miR-145-5p could partially reverse the proliferation-inhibitory effect of siRNA-hsa_circ_101555 (Fig. 5I–L). Moreover, the decrease in HepG2 and HCCLM3 cell migration and invasion caused by hsa_circ_101555 silencing could be reversed by miR-145-5p inhibition (Fig. 5M–N). Collectively, these results demonstrate that hsa_circ_101555 was required to sustain HCC cell progression and partly functions by impairing the tumor suppressor miR-145-5p.

### Analysis of CDCA3 expression in HCC

**Fig. 6:**
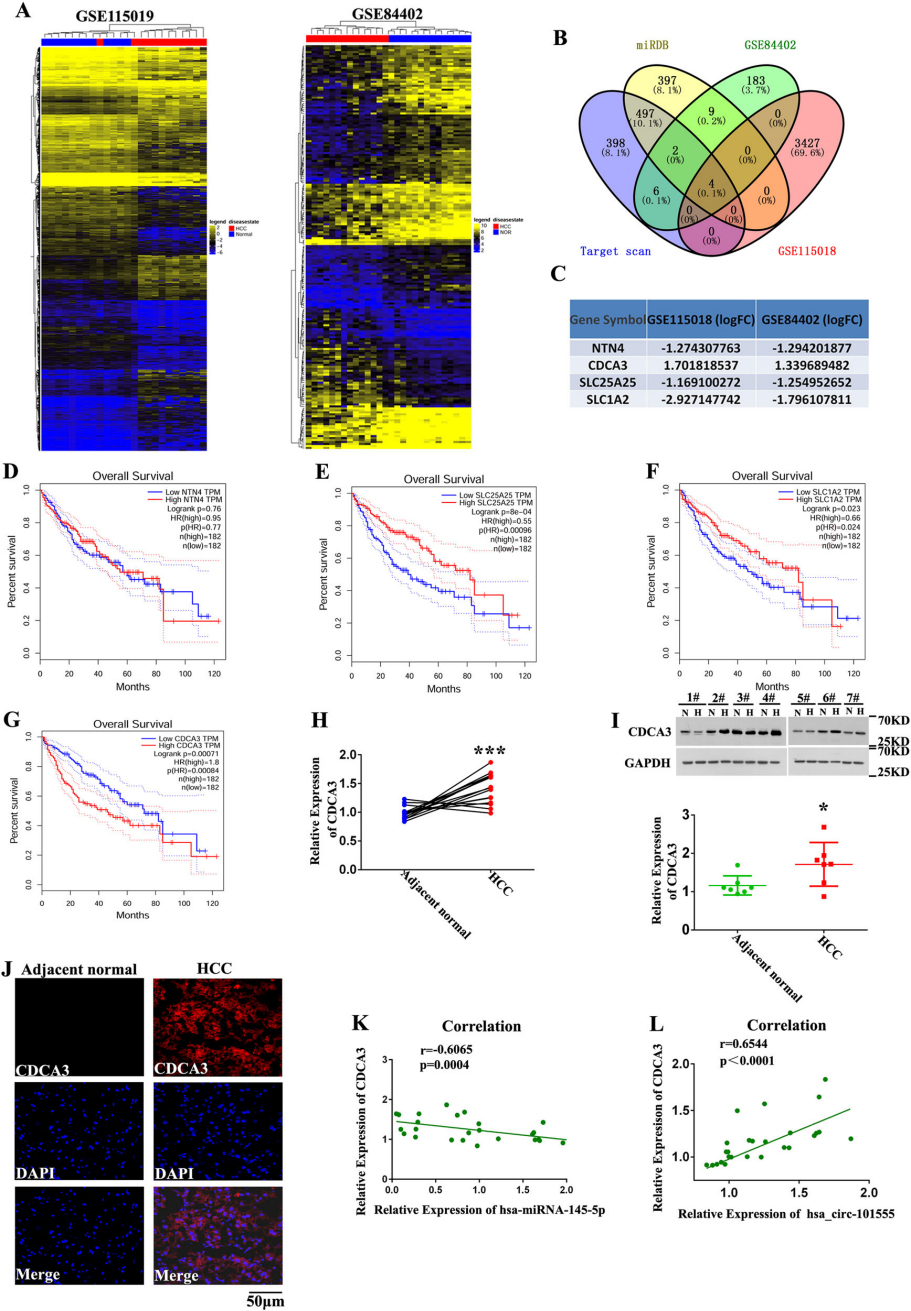
Identification of *CDCA3* as an miRNA target oncogenes. **A** Heatmap analyses demonstrating differentially expressed mRNAs in HCC with |log2fold-change | > 1.0 and *P* < 0.05. **B**, **C** Venn analysis between predicted mRNA genes targeted by miR-145-5p and differentially expressed mRNAs identified four mRNAs, *NTN4, CDCA3, SLC25A25*, and *SLC1A2* involved in HCC. **D**–**G** *CDCA3* is significantly upregulated in HCC tumors and is associated with poorer OS. **H** Expression of *CDCA3* between ten HCC tumor tissues and adjacent normal tissues measured by qRT-PCR (***P < 0.001, *n* = 10). **I** Expression of CDCA3 between seven HCC tumor and adjacent normal tissues measured by western blot (**P < 0.01; *n* = 7). **J** CDCA3 protein expression in HCC and corresponding adjacent normal tissues detected by IHC (*n* = 3). **K** Pearson correlation analysis demonstrating correlation between miR-145-5p and CDCA3 expression (*n* = 20). **L** Pearson correlation analysis showing the correlation between hsa-circ-101555 and CDCA3 expression (*n* = 20).

To better understand the regulatory mechanism of miR-145-5p in HCC, we identified its potential target genes using mRNA array from GSE115019 and GSE84402 datasets (Fig. 6A). Potential miR-145-5p target mRNAs were predicted by bioinformatics (TargetScan and miRanda). We then identified four miR-145-5p-related genes (*NTN4, CDCA3, SLC25A25*, and *SLC1A2*) by Venn analysis between HCC-related and miR-145-5p predicted genes (Fig. 6B and C). Analysis of the overall survival of these miR-145-5p-related genes using GEPIA (http://gepia.cancer-pku.cn/) revealed that high *CDCA3* levels were associated with poorer overall survival (Fig. 6D–G).

We further detected *CDCA3* mRNA expression in ten paired HCC and adjacent normal tissues by qRT-PCR to better understand its role and found it to be significantly upregulated in HCC tumors (Fig. 6H). Additionally, western blot and immunohistochemistry (IHC) analyses revealed that the protein expression of CDCA3 in HCC tissues was significantly upregulated as compared to their adjacent normal tissues (Fig. 6I and J).

These results demonstrate that CDCA3 may represent a primary target of miR-145-5p. Pearson correlation analysis further revealed a negative correlation (*p* = 0.0004, r = −0.6065) between miR-145-5p and CDCA3 expression levels (Fig. 6K) and a positive correlation (*p* < 0.0001, r = 0.6544) between hsa_circ_101555 and CDCA3 expression levels (Fig. 6L).

### miR-145-5p directly targets the CDCA3 3ʹ-UTR

**Fig. 7:**
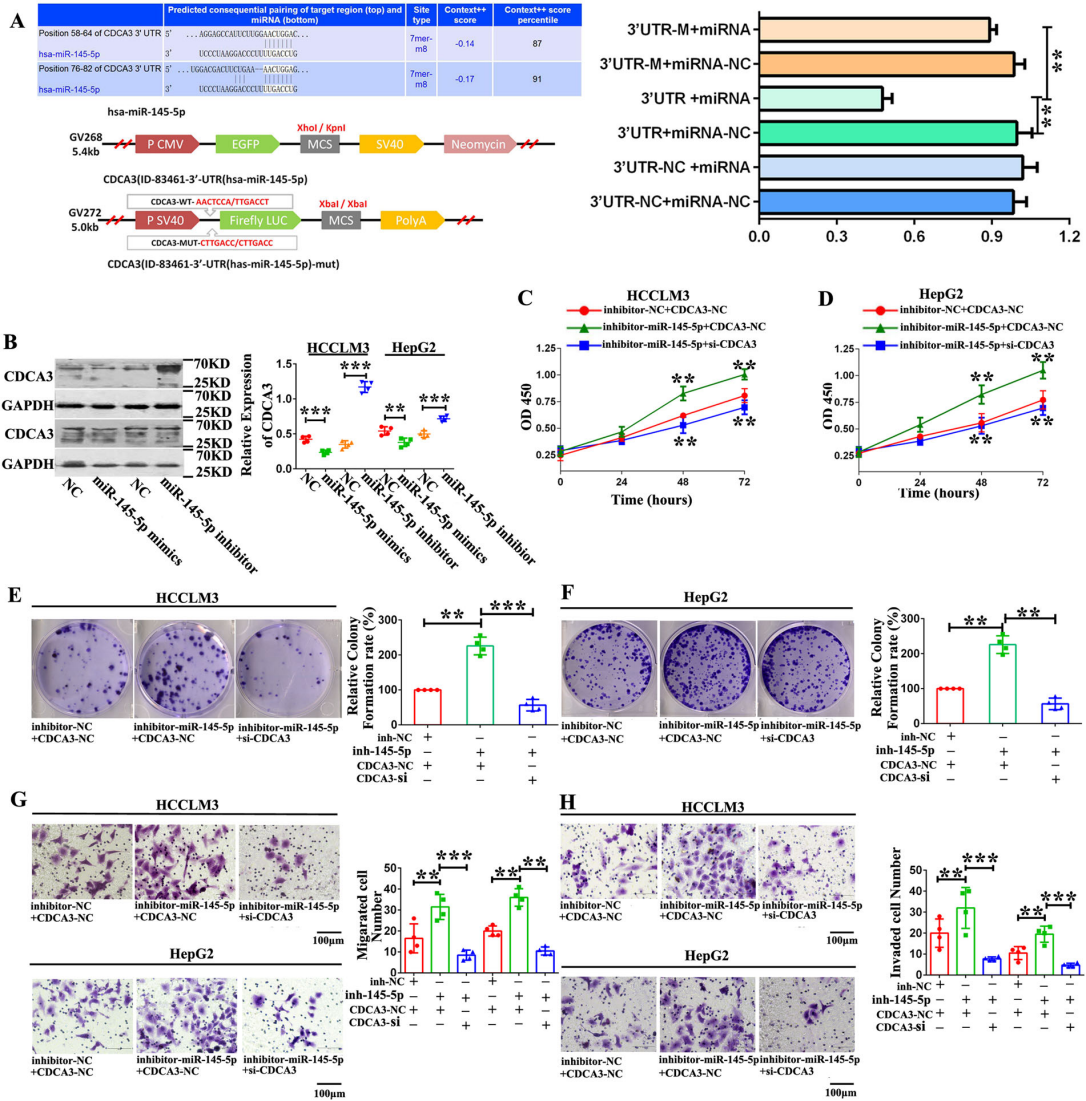
miR-145-5p targets CDCA3 to sustain HCC cell tumorigenicity. **A** Schematic of CDCA3 3′ UTR wild-type (WT) and mutant (Mut) luciferase reporter vectors. Relative luciferase activities analyzed in 293 T cells co-transfected with the miR-145-5p plasmid or miR-NC and luciferase reporter vectors CDCA3 3′ UTR (WT) or CDCA3 3′ UTR (Mut) (*P < 0.05; *n* = 3). **B** Western blot analysis indicating that miR-145-5p downregulates CDCA3 (**P < 0.01; ***P < 0.001; *n* = 4). **C**, **D** HCCLM3 and HepG2 cell viability following co-transfection with siRNA-CDCA3 and miR-145-5p inhibitor, measured by WST-1 assays (**P < 0.01; *n* = 4). **E**, **F** Proliferative capacity of HCCLM3 and HepG2 cells co-transfected with siRNA-CDCA3 and miR-145-5p inhibitor, evaluated by colony formation assay (**P < 0.01; ***P < 0.001; *n* = 4). **G**, **H** The influence on cell migration and invasion of HCCLM3 and HepG2 cells transfected with siRNA-CDCA3 and miR-145-5p inhibitor, assessed by transwell migration and Matrigel invasion assays (**P < 0.01; ***P < 0.001; *n* = 4).

The miR-145-5p–CDCA3 interaction was confirmed via luciferase reporter assays, where in miR-145-5p significantly reduced the activity of the luciferase reporter compared to the negative control (wild-type CDCA3 sequence). However, such reduction was not observed following mutation of the miR-145-5p binding sites (Fig. 7A). These results indicate that miR-145-5p negatively regulated the expression of CDCA3.

Moreover, miR-145-5p inhibition significantly increased CDCA3 protein levels, and miR-145-5p mimetic agents reduced CDCA3 expression in HepG2 and HCCLM3 cells (Fig. 7B). Meanwhile, knockdown of hsa_circ_101555 significantly inhibited *CDCA3* expression, while overexpression of hsa_circ_101555 had the opposite effect (Supplementary Fig. 7A and B). Additionally, downregulation of *CDCA3* in HepG2 and HCCLM3 cells, caused by hsa_circ_101555 silencing, could be reversed by miR-145-5p inhibition (Supplementary Fig. 7C). miR-145-5p inhibition also promoted the proliferation, migration, and invasion of HCC cells. Importantly, these enhancements were not observed in cells co-transfected with siRNA-CDCA3 and miR-145-5p inhibitor (Fig. 7C–H), suggesting that miR-145-5p targets CDCA3 and subsequently inhibits the proliferation, migration, and invasion of HCC cells.

### hsa_circ_101555 is required to sustain HCC tumor growth in vivo

**Fig. 8:**
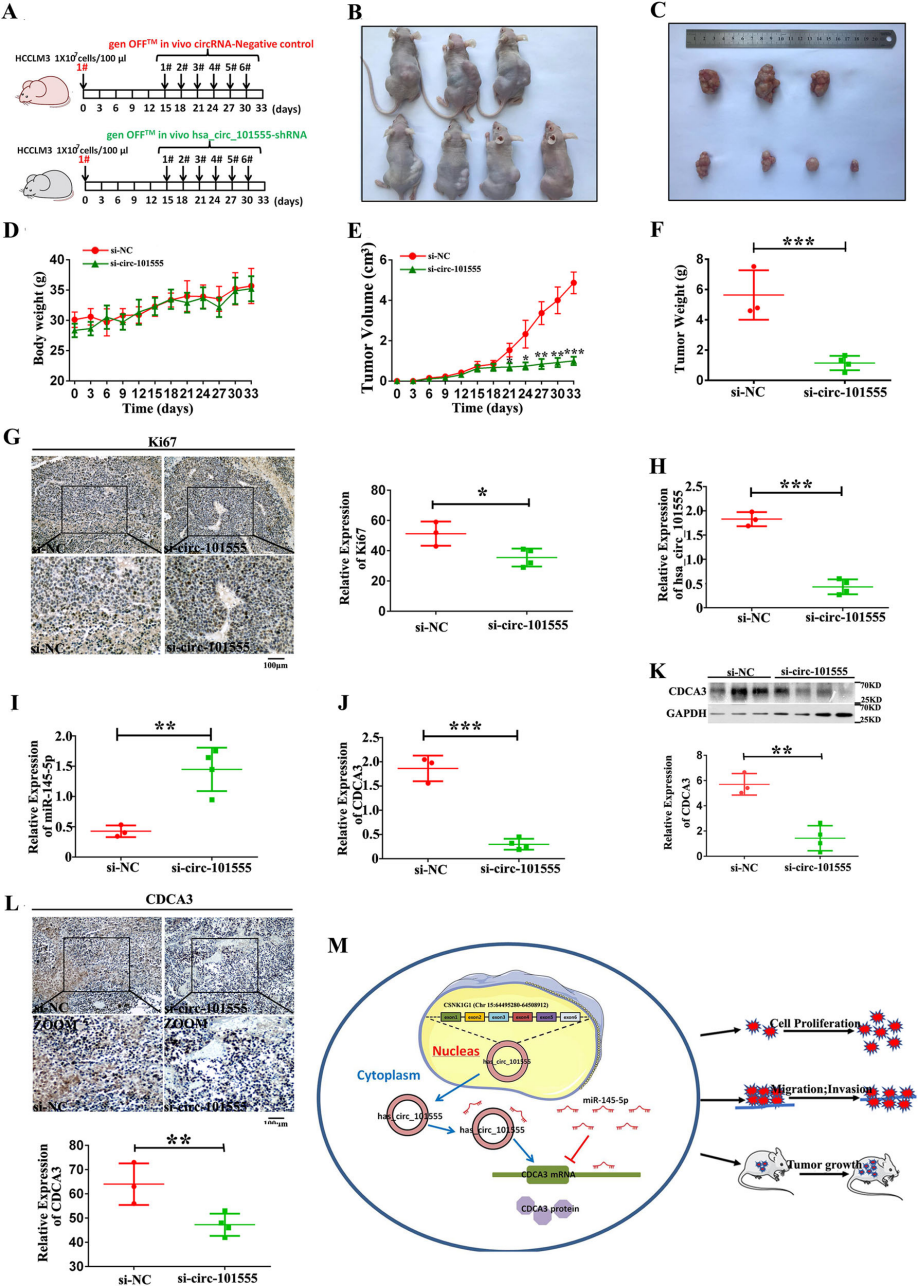
hsa_circ_101555 promotes HCC tumor growth in vivo. **A** Schematic diagram shows that HCCLM3 cells were injected subcutaneously into BALB/c nude mice for 15 days to create tumor xenografts. After 15 days, 10 nmol cholesterol-modified hsa_circ_101555 shRNA or negative control shRNA was subcutaneously injected at the tumor site every 3 days for 18 days. **B** Representative images of HCC tumor bearing BALB/c nude mice. **C** Body weight of xenograft nude mice. **D** Body weight measured every 3 days. **E** Tumor volumes measured every 3 days (*P < 0.05; **P < 0.01; ***P < 0.001; *n* = 4). **F** Relative weights of tumors (***P < 0.001; *n* = 4). **G** IHC staining and IHC analysis of Ki-67 expression in subcutaneous xenograft tumors. Knockdown of hsa_circ_101555 downregulates Ki-67 expression (*P < 0.05; *n* = 4). **H**–**J** Expression of hsa_circ_101555, miR-145-5p and CDCA3 in subcutaneous xenograft tumors (**P < 0.01; ***P < 0.001; *n* = 4). **K** Protein expression level of CDCA3 in seven paired HCC and normal tissues (**P < 0.01; *n* = 4). **L** IHC staining and IHC analysis of CDCA3 expression in subcutaneous xenograft tumors. Knockdown of hsa_circ_101555 downregulates CDCA3 expression (**P < 0.01; *n* = 4). **M** Schematic diagram shows that hsa_circ_101555 promotes HCC cells proliferation, migration, and invasion through the miR-145-5p/CDCA3 axis.

Considering that study demonstrated that hsa_circ_101555 knockdown suppressed the viability of HepG2 and HCCLM3 cells, we further examined its role in HCC tumorigenesis in vivo. Figure 8A provides a schematic diagram of tumor xenograft generation and subsequent treatment with cholesterol-modified hsa_circ_101555 shRNA or negative control shRNA. Body weight and tumor volume measurements, taken every 3 days for 33 days, revealed that mice receiving hsa_circ_101555 shRNA exhibited a marked decrease in tumor volume and weight compared to those receiving control shRNA (Fig. 8B–F). This suggested that hsa_circ_101555 promoted HCC cell growth in vivo. These results further supported a role for hsa_circ_101555 in HCC tumorigenesis and development.

Moreover, immunohistochemical staining of xenografted tumors demonstrated that hsa_circ_101555 silencing significantly reduced the Ki-67 proliferation index (Fig. 8G). Hsa_circ_101555 silencing also significantly inhibited hsa_circ_101555 and CDCA3 expression, while increasing miR-145-5p expression in the hsa_circ_101555-silenced group compared to the negative control group (Fig. 8H–J). Western blotting further demonstrated that hsa_circ_101555 silencing significantly reduced CDCA3 abundance (Fig. 8K), which was supported by immunohistochemical staining of xenografted tumors (Fig. 8L).

## Discussion

In the present study we identified 36 circRNAs with differential and consistent expression in GSE94508, GSE97322, and GSE78520 datasets. As hsa_circ_101555 was the only upregulated circRNA appearing in the three GSE datasets, it became the focus of further analyses. We characterized hsa_circ_101555 in HCC cells, and found that it consisted of six exons (exon 1–6, 815 nt) and was derived from the host gene *CSNK1G1*. Moreover, it was cyclized with the head-to-tail splicing of exon 1 and exon 6 and resisted RNase R treatment, demonstrating its stability compared to linear RNA.

Previous studies have shown that circRNAs play an essential role in cell cycle progression and proliferation^26–29^. hsa_circ_101555 was specifically reported as upregulated in tumor tissues and as associated with the prognosis of colorectal cancer patients, while its silencing significantly suppresses cell proliferation, induces apoptosis, and impairs the DNA repair capacity of CRC cells^30^. Herein, we found that hsa_circ_101555 was highly expressed in HCC cell lines (most markedly in HCCLM3) as well as patient tissues compared to adjacent nontumor tissues. More importantly, in a murine xenograft model, hsa_circ_101555 silencing significantly reduced HCC growth. We also provided evidence that the ectopic expression of hsa_circ_101555 is likely required to sustain cell proliferation. Meanwhile, we previously demonstrated that circMAST1 silencing inhibits HCC cell migration and invasion, which are important determinants of tumor metastasis^31^. Our results are consistent with those of previous studies that showed a regulatory role for circRNAs in cancer proliferation, migration, and invasion^32–36^. Thus, our research confirmed the stable role of hsa_circ_101555 in promoting HCC progression. Although the mechanisms through which circRNAs regulate carcinogenesis and cancer progression have not been fully elucidated, the “circRNA–miRNA–mRNA” axis, also known as the “miRNA sponge,” is frequently cited^37^. In the present study, we confirmed that hsa_circ_101555 is an miR-145-5p sponge, evidenced by the significant increase in miR-145-5p expression following silencing of hsa_circ_101555, which in turn inhibited the proliferation, migration, and invasion of HCC cell lines. We also confirmed a direct correlation between miR-145-5p and hsa_circ_101555 expression. Consistent with our findings, several other studies have shown that circRNAs act as miRNA sponges during the development and progression of HCC. Hu et al. reported that circASAP1 acts as a ceRNA for miR-326 and miR-532-5p, which are tumor suppressors that regulating cancer cell proliferation, colony formation, migration, and invasion^38^. Further, many miRNAs have been shown to play critical roles in HCC initiation, development, and progression^39–43^.miR-145-5p is a tumor suppressor that is downregulated in several cancer types including glioma^44^, upper tract urothelial carcinoma^45^, and gastric cancer^46^. Consistent with these reports, our findings indicate that miR-145-5p serves as a tumor suppressor miRNA in HCC. As hsa_circ_101555 sponges miR-145-5p, the increased expression of hsa_circ_101555 in HCC cells leads to a decrease in miR-145-5p expression, thereby promoting cancer cell proliferation, migration, and invasion. Conversely, inhibiting hsa_circ_101555 expression increased miR-145-5p, which consequently suppressed the proliferation, migration, and invasion of HCC cells. However, we found that simultaneous inhibition of both hsa_circ_101555 and miR-145-5p expression, resulted in increased tumorigenic properties in HCC cells compared to that in cells with only hsa_circ_101555 inhibition. Thus, our results provide evidence that hsa_circ_101555 regulates HCC progression via miR-145-5p sponging, and that hsa_circ_101555 is an upstream target of miR-145-5p.

The role of miRNA sponging in tumor progression has previously been described^47^. We confirmed, via bioinformatics and luciferase reporter gene analyses, that hsa_circ_101555–miR-145-5p targets the oncogene *CDCA3*. Although several studies have implicated *CDCA3* in the regulation of cancer development and progression^48–50^, to our knowledge, this is the first study to confirm *CDCA3* expression in liver cancer tissues and report its positive correlation with hsa_circ_101555 expression and negative correlation with miR-145-5p expression. Meanwhile, a previous study in colorectal cancer demonstrated that miR-145-5p suppressed proliferation, metastasis, and epithelial–mesenchymal transition by targeting *CDCA3*^51^. Our results were consistent with these reports. In addition, we found that the miR-145-5p/CDCA3 axis is regulated by hsa_circ_101555 via a sponging mechanism. Further, we demonstrated that miR-145-5p suppression promotes CDCA3 expression, which in turn increases the proliferation, migration, and invasion of HCC cells. The simultaneous inhibition of miR-145-5p and *CDCA3* attenuates the tumorigenic features of HCC cells to a greater extent than miR-145-5p inhibition alone. To our knowledge, our study is the first to demonstrate hsa_circ_101555 involvement in regulation of *CDCA3* expression. Moreover, we demonstrated that hsa_circ_101555 silencing significantly reduces *CDCA3* expression in vivo. These findings suggest that hsa_circ_101555 protects CDCA3 from miR-145-5p-mediated degradation in a ceRNA-mediated manner.

The biogenesis of circRNAs is regulated by specific cis-acting elements and trans-acting factors. It has been shown that certain RNA-binding proteins promote circRNA expression^52^. RNA-binding protein EIF4A3 is the core component of exon junction complex (EJC), which is considered as an important regulator of post-transcriptional regulation processes including mRNA splicing, transport, translation, and surveillance^53^. Through bioinformatic analysis and experiments, we predicted and screened that EIF4A3 could bind to a flanking sequence of hsa_circ_101555. Our research reveals that EIF4A3-mediated reverse splicing of exons can be a potential mechanism to induce high expression of hsa_circ_101555.

We acknowledge that our research has certain limitations. Although this study clarifies that hsa_circ_101555 functions as a sponge of miR-145-5p to promote CDCA3-induced HCC cancer cell proliferation and invasion, circRNAs may regulate the development and progression of HCC via other mechanisms. For example, circRNAs have been shown to regulate parental gene expression and the expression of peptides/proteins in other cancers^36,54^. Thus, additional research is required to further explore the role of hsa_circ_101555 in HCC. In terms of clinical diagnosis and treatment, we additional studies are required, including expanding the sample size and expression stability of hsa_circ_101555 in the peripheral blood of HCC patients, as well as evaluating the initiation of its high expression among the stages of HCC.

To summarize, we found that: (1) hsa_circ_101555 is highly expressed in HCC tissues and HCC cell lines (HepG2 and HCCLM3); (2) silencing hsa_circ_101555 in a murine xenograft model significantly reduces the growth of HCC; (3) hsa_circ_101555 is likely required to sustain proliferation, migration, and invasion in HCC cell lines; (4) hsa_circ_101555 acts as an miR-145-5p sponge, while silencing hsa_circ_101555 significantly inhibits cell growth; (5) hsa_circ_101555 sponges miR-145-5p to promote CDCA3 expression; and (6) eIF4A3 induces hsa_circ_101555 cyclization and increases hsa_circ_101555 expression. Thus, our study identified a previously unrecognized role for hsa_circ_101555 in sustaining HCC progression.

## Conclusions

Our study demonstrated that hsa_circ_101555 is upregulated in HCC cell lines and patient tissues, and its high expression is associated with HCC progression. Moreover, hsa_circ_101555 functions as a tumor promotor and is required to sustain the proliferation and invasion of HCC by directly binding to miR-145-5p and impeding its suppression of CDCA3. Furthermore, we also demonstrate that EIF4A3 could mediate the biogenesis of hsa_circ_101555, but the detailed mechanism needs further study. Based on its role in regulating the miR-145-5p/CDCA3 axis, our findings suggest that hsa_circ_101555 may represent a potential novel biomarker and therapeutic target for HCC.

## Materials and methods

### Gene Expression Omnibus dataset

We downloaded the expression microarray data (CEL data) from the GSE7852, GSE94508, GSE97322, GSE115016, GSE41874, GSE115018, and GSE84402 dataset of the GEO (http://ncbi.nlm.nih.gov/geo/). Detailed expression profiles are provided in Additional file 1–7.

### Cell lines and clinical tissues

A total of 38 HCC samples were obtained from the clinical sample bank of the First Affiliated Hospital, Harbin medical university. The collection of human specimens was approved by the Biomedical Ethics Committee of the Harbin medical university First Affiliated Hospital and written informed consent was obtained from each patient (HMUDQ20200010202). Inclusion criteria for patient selection was curative hepatectomy performed between 2017 and 2018. All patients were pathologically diagnosed with hepatocellular carcinoma and liver specimens were evaluated by pathologists to determine clinical staging according to the TNM classification. HCC patients with the following conditions were excluded: (1) patients ≤18 or ≥70 years of age or without full civil capacity; (2) patients with a history of preoperative anticancer radiotherapy or chemotherapy, biological, immune, or traditional Chinese medicine administration; (3) patients with incomplete postoperative follow-up data; (4) patients with a history of another organ malignancy, or systemic immune disease. All specimens were collected within 15 min of removal from the body and were immediately snap-frozen in liquid nitrogen before storage at −80 °C. Ten paired samples were used to compare the expression levels of the genes of interest between HCC and paired nontumorous tissues. The detailed clinicopathological features, as well as the correlations between hsa_circ_101555 expression and the clinical characteristics, are described in Additional files 8–9: Tables S1-S2.

Cell lines used in this study (HepG2, HCCLM3, and SK-Hep1) were purchased from the Cell Bank of Type Culture Collection (Chinese Academy of Sciences, Shanghai, China). Huh-7 and L02 were purchased from the Procell (Wuhan, China). All cells were cultured in DMEM/high glucose medium (Hyclone, Logan, UT, USA) supplemented with 10% fetal bovine serum (PAN-Biotek, Aidenbach, Bavaria) and 1% penicillin-streptomycin (Hyclone) in a humidified atmosphere at 37 °C containing 5% CO_2_.

### Xenograft nude mouse model

Six-week-old male BALB/C nude mice purchased from Vital river (Beijing, China) were maintained under specific pathogen-free conditions with a 12-h light/dark cycle. All animal experiments were performed in accordance with a statement of compliance with ethical regulations and approved by the Biomedical Ethics Committee of the Harbin medical university. Animals are grouped randomly during the experiment. HCCLM3 cells were subcutaneously injected into the right upper back of the nude mice (1 × 10^6^ cells per mouse) for 15 days, after subcutaneous incubation of HCC tumor mass. Next, 10 nmol cholesterol-modified hsa_circ_101555 shRNA or control shRNA RiboBio (Guangzhou, China) were intratumorally injected every 3 days for 18 days. the last day of the injections, which would account for day 33 of the experiment, the mice were sacrificed and tumor tissues were collected for examination.

### Circular structure confirmation

The circular structure of hsa_circ_101555 was confirmed by Sanger sequencing, divergent primer PCR and RNase R treatment. PCR products, amplified by divergent primers of hsa_circ_101555, were inserted into the T vector and delivered to SinoGENE (Beijing, China) for Sanger sequencing. The results were crosschecked with the back-spliced region of hsa_circ_101555 supplied by circBASE^55^. Total RNA was extracted from HCC tissue using Trizol solution. For RNase R treatment, 3 μg of total RNA extracted from HCC was incubated with 10 U RNase R (20 U/μL, Epicentre, Madison, WI, USA) in a 10 μL total volume at 37 °C for 45 min, followed by incubation at 70 °C for 10 min to deactivate RNase R. The treated RNAs were used for qRT-PCR^27^.

### Quantitative real-time polymerase chain reaction, western blotting, and immunofluorescence analyses

Total RNA was extracted from HCC cell lines and tissue using Trizol solution, and complement DNA was generated using the Golden 1st cDNA Synthesis kit (Haigene, China) following RNA quantification. qRT-PCR assays were performed using Power SYBB Green PCR Master Mix (Life Technologies, Carlsbad, CA, USA). The circRNA and gene expression levels were normalized to that of *GADPH*, while miRNA expression levels were normalized to that of U6. Each sample was tested in triplicate. The relative expression was analyzed by the comparative cycle threshold (Ct) method, according to the equation 2^−ΔΔCt^ [Δ^Ct^ = Ct-Ct (GAPDH)]. The primer sequences of hsa_circ_101555 and U6 were designed by RiboBio (Guangzhou, China). The linear101555, CDCA3, miR-145-5p, and GAPDH sequences were designed by Genscript (Nanjing, China). All experiments were performed in triplicate. The primers used in this study are listed in Additional file 10: Table S3. RNase R digestion was performed using RNase R purchased from Epicentre Biotechnologies (Madison, WI, USA) as per the manufacturer’s instructions. Briefly, 1 µg of extracted RNA was treated with 3 U of RNase R (Epicenter, USA) and incubated at 37 °C for 1 h, after which the RNA was purified by phenol-chloroform extraction and subjected to qRT-PCR.

For western blotting, the total protein extracts from cells were separated by sodium dodecyl sulfate-polyacrylamide gel electrophoresis (SDS-PAGE), transferred onto polyvinylidene difluoride membranes, and incubated with the corresponding antibodies. The membranes were developed using the enhanced chemiluminescence method (Haigene, China). All antibodies used in this study are listed in Additional file 11: Table S4.

Fresh samples were cut to an appropriate size and fixed in 4% paraformaldehyde for 24 h. The fixed specimens were dehydrated in a graded series of ethanol solutions, embedded in paraffin and cut at a thickness of 4 μm. The sections were dewaxed and rehydrated using xylene and ethanol, and high-pressure heat was applied for antigen retrieval. The sections were incubated with the primary antibodies overnight at 4 °C. Finally, all sections were dehydrated, cleared, mounted, and visualized with a diaminobenzidine-based colorimetric method. The antibodies used in this study are listed in Additional file 11: Table S4.

### Fluorescence in situ hybridization

In situ hybridization was performed with a FISH kit (RiboBio, Guangzhou, China). Cells, frozen HCC sections, and paired adjacent liver tissues were briefly rinsed in PBS and fixed in 4% formaldehyde for 10 min. The cells were then permeabilized in PBS containing 0.5% Triton X 100 at 4 °C for 5 min, washed with PBS three times for 5 min, and prehybridized at 37 °C for 30 min before hybridization. Next, an anti- hsa_circ_101555, anti-U6, or anti-18S oligodeoxynucleotide probe (RiboBio, Guangzhou, China) was used in the hybridization solution at 37 °C overnight in the dark. The next day, cells were counterstained with DAPI and imaged using a NA1.4 inverted Leica DMI6000 microscope (Leica, Heidelberg, Germany). Images were captured using a Hamamatsu ORCA-R2 camera (Hamamatsu, Japan) and recorded using LAS AF software (Leica). The experiments were conducted in triplicate.

### Cell proliferation, migration, and Matrigel invasion assay

Cell proliferation was assessed using the WST-1 assay (Beyotime Biotechnology, Nantong, China). Cells (2 × 10^3^) were seeded into each well of 96-well plates and 10 μL of WST-1 solution was added to each well at four timepoints (0, 24, 48, and 72 h). After 4 h of incubation at 37 °C, the absorbance at 450 nM was measured using a Spectra Max 250 spectrophotometer (Molecular Devices, Sunnyvale, CA, USA). Experiments were independently performed in triplicate.

For the colony formation assays, cells (1 × 10^2^) were suspended and plated into each well of 6-well plates. After 14 days incubation at 37 °C in a chamber with an atmosphere of 5% CO_2_, colonies were fixed with 1 mL of 4% paraformaldehyde (Solarbio, Beijing, China) for 30 min and stained with crystal violet (Beyotime Biotech-nology, Nantong, China) for 25 min. Colonies were then counted after photographing the sample (Nikon, Tokyo, Japan).

Cell migration and invasion were measured using a transwell migration assay and a Matrigel invasion assay. For the transwell migration assay, 2–4 × 10^5^ cells were suspended in 200 µL of DMEM without serum and placed in the cell culture insert (8 µm pore size; BD Falcon, San Jose, CA) of a companion plate (BD Falcon) with prewarmed culture medium containing 20% fetal bovine serum. The cells were incubated for 24 h at 37 °C in 5% CO_2_ and subsequently fixed with 4% paraformaldehyde in PBS.

For the Matrigel invasion assay, 2–4 × 10^5^ cells were suspended in 200 µL of DMEM without serum and were placed in the cell culture insert precoated with 50 µL Matrigel (BD Biosciences, San Jose, CA, USA). A prewarmed culture medium containing 20% fetal bovine serum was added to the well. The cells were incubated for 48 h at 37 °C in 5% CO_2_ and were then fixed with 4% paraformaldehyde in PBS. The nonmigrated or invaded cells on the top of the membrane were gently removed with a cotton swab. Cell migration or invasion was determined by staining cells with 0.1% crystal violet (Sigma, St Louis, MO) and the cells were counted under a light microscope (×200 magnification) in eight randomly selected areas.

### Transfection experiment

siRNA specific for hsa_circ_101555 was synthesized by RiboBio (Guangzhou, China), while the inhibitor of the miR-145-5p mimics and negative control, miRNA-145-5p, siRNA of CDCA3 were synthesized by Gene Pharma (Shanghai, China). HepG2 and HCCLM3 cells were transfected with siRNA of hsa_circ_101555 or Huh-7 and SK-hep1 cells were transfected with lv-hsa_circ_101555 using the Lipofectamine 2000® siRNA transfection reagent (Invitrogen, Carlsbad, CA, USA) following the manufacturer’s protocol. The target sequences of siRNAs are listed in Additional file 12: Table S5.

### Dual luciferase assay

Targeted binding of hsa_circ_101555 to miR-145-5p was predicted using bioinformatics websites, including CircInteractome, and miRanda; whereas targeted binding of miR-145-5p to CDCA3 was predicted using TargetScan, and miRDB. The full-length sequences of hsa_circ_101555, with and without mutated predicted miR-145-5p binding sites, were subcloned into pmirGLO reporter vector (Promega, WI, USA). The full-length sequences of CDCA3, with and without mutated predicted miR-145-5p binding sites, were subcloned into pmirGLO reporter vector (Promega, WI, USA). Lipo2000 was then used for transfection of the vectors into 293 T cells. Finally, luciferase activity was measured using the dual luciferase assay kit.

### RNA pull-down

RNA pull-down technology used desulphurized biotin-labeled RNA of *CSNK1G1* mRNA and streptavidin-labeled magnetic beads to efficiently enrich and identify the RNA-binding protein, EIF4A3. RNA probes were labeled with biotin by in vitro transcription, and then incubated with cytoplasmic protein extract to form RNA-protein complexes. This complex was then allowed to bind streptavidin-labeled magnetic beads to separate them from the other components in the solution. After elution, western blotting was used to detect the binding proteins of EIF4A3 that interacted with *CSNK1G1* mRNA.

### RIP assay

RIP (RNA-binding protein immunoprecipitation) assay was performed with the BersinBio™ RNA Immunoprecipitation kit (Guangzhou, China). Cells were collected and cultivated in RIP lysis buffer, followed by immunoprecipitation with EIF4A3 antibody (Abcam, MA, USA). The final retrieved RNA was subjected to quantitative real-time PCR analysis. Normal mouse immunoglobulin G (IgG) served as negative controls.

### Statistical analyses

All statistical analyses were performed using SPSS version 21.0 (IBM SPSS Inc., Chicago, IL, USA) and GraphPad Prism version 6.0 (GraphPad Software, La Jolla, CA, USA) software. Categorical variables are expressed as a count or percentage and tested using Chi-squared or Fisher’s exact tests, as appropriate. Continuous data are reported as mean ± standard deviation (SD) and compared using Student’s *t*-test, one-way analysis of variance (ANOVA) test, or Mann–Whitney U-test, as appropriate. Correlations were calculated using Pearson correlation analysis. *P* < 0.05 was considered statistically significant.

## Supplementary information

Supplement Materials and Methods-Additional file 1 GSE78520

Supplement Materials and Methods-Additional file 2 GSE94508

Supplement Materials and Methods-Additional file 3 GSE97332

Supplement Materials and Methods-Additional file 4 GSE115016

Supplement Materials and Methods-Additional file 5 GSE41874

Supplement Materials and Methods-Additional file 6 GSE115018

Supplement Materials and Methods-Additional file 7 GSE84402

Supplement Materials and Methods-Additional file 8 Table S1

Supplement Materials and Methods-Additional file 9 Table S2

Supplement Materials and Methods-Additional file 10 Table S3

Supplement Materials and Methods-Additional file 11 Table S4

Supplement Materials and Methods-Additional file 12 Table S5

SUPPLEMENTAL MATERIALSupplementary Figure Legends

Supplemental Figure 1

Supplemental Figure 2

Supplemental Figure 3

Supplemental Figure 4

Supplemental Figure 5

Supplemental Figure 6

Supplemental Figure 7

Supplemental Figure 8

Supplemental Figure 9
