# Supplementary material for: Circular RNA hsa_circ_101555 promotes hepatocellular carcinoma cell proliferation and migration by sponging miR-145-5p and regulating CDCA3 expression
Source: Cell Death Dis. 2021 Apr 6;12(4):356. doi: 10.1038/s41419-021-03626-7 (PMC8024300; doi:10.1038/s41419-021-03626-7)
Supplement: Supplementary file 8 — Supplement Materials and Methods-Additional file 8 Table S1 [file 41419_2021_3626_MOESM8_ESM.docx]

**Additional file 8: Table S1. The clinicopathological characterization details of 38 HCC patients.**

| Patient number | Age | Gender | TNM | Pathological stage |
| --- | --- | --- | --- | --- |
| 1 | 56 | Male | T2N0M0 | Ⅱ |
| 2 | 62 | Male | T2N0M0 | Ⅱ |
| 3 | 51 | Male | T2N0M0 | Ⅱ |
| 4 | 59 | Male | T3bN0M0 | Ⅱ |
| 5 | 60 | Female | T2N1M0 | Ⅱ |
| 6 | 59 | Male | T2N0M0 | Ⅱ |
| 7 | 52 | Male | T4N1M0 | Ⅳ |
| 8 | 58 | Male | T3aN0M0 | Ⅱ |
| 9 | 46 | Male | T3aN0M0 | Ⅲ |
| 10 | 46 | Male | T2N0M0 | Ⅱ |
| 11 | 38 | Female | T3aN0M0 | Ⅲ |
| 12 | 50 | Male | T2N0M0 | Ⅰ |
| 13 | 65 | Male | T3aN0M0 | Ⅲ |
| 14 | 46 | Male | T3aN0M0 | Ⅲ |
| 15 | 59 | Male | T3aN0M0 | Ⅲ |
| 16 | 53 | Male | T2N0M0 | Ⅱ |
| 17 | 54 | Male | T3aN0M0 | Ⅱ |
| 18 | 45 | Male | T3aN0M0 | Ⅳ |
| 19 | 57 | Male | T2N0M0 | Ⅱ |
| 20 | 64 | Male | T1N0M0 | Ⅰ |
| 21 | 64 | Male | T1N0M0 | Ⅱ |
| 22 | 35 | Male | T2N0M0 | Ⅱ |
| 23 | 57 | Male | T2N0M0 | Ⅲ |
| 24 | 59 | Male | T2N0M0 | Ⅱ |
| 25 | 75 | Male | T2N0M0 | Ⅱ |
| 26 | 66 | Male | T1N0M0 | Ⅱ |
| 27 | 59 | Male | T2N0M0 | Ⅱ |
| 28 | 51 | Male | T2N0M0 | Ⅲ |
| 29 | 54 | Male | T2N0M0 | Ⅱ |
| 30 | 68 | Male | T2N0M0 | Ⅲ |
| 31 | 52 | Male | T2N0M0 | Ⅳ |
| 32 | 59 | Male | T1N0M0 | Ⅳ |
| 33 | 45 | Female | T2N0M0 | Ⅱ |
| 34 | 68 | Male | T2N0M0 | Ⅱ |
| 35 | 67 | Female | T1N0M0 | Ⅱ |
| 36 | 57 | Male | T2N0M0 | Ⅱ |
| 37 | 48 | Male | T4N0M0 | Ⅳ |
| 38 | 52 | Male | T4N0M0 | Ⅲ |
