# Supplementary material for: Circular RNA hsa_circ_101555 promotes hepatocellular carcinoma cell proliferation and migration by sponging miR-145-5p and regulating CDCA3 expression
Source: Cell Death Dis. 2021 Apr 6;12(4):356. doi: 10.1038/s41419-021-03626-7 (PMC8024300; doi:10.1038/s41419-021-03626-7)
Supplement: Supplementary file 9 — Supplement Materials and Methods-Additional file 9 Table S2 [file 41419_2021_3626_MOESM9_ESM.docx]

**Additional file 9: Table S2. Clinical Characteristics of 38 HCC Patients According to hsa_circ_101555 Expression Level**

| **Variable** | **hsa_circ_101555**  **High Low** | **P-value** |
| --- | --- | --- |
| All cases | 26 12 |  |
| Age, years,>50: ≤50 | 18:8 11:1 | 0.223206 |
| Gender, male/female | 24:2 10:2 | 0.577322 |
| HBsAg, positive/negative | 20:6 0:12 | 0.000007*** |
| AFP, µg/L,>400: ≤400 | 9:17 3:9 | 0.714374 |
| Tumour size, cm, >3: ≤3 | 24:2 10:2 | 0.577322 |
| Microvascular invasion, yes:no | 23:3 10:2 | 0.642588 |
| Lymphonodemetastasis, yes:no | 1:25 1:11 | 0.537696 |
| TNM stage, I ~ II: III ~Ⅳ | 12:14 12:0 | 0.001104** |

χ^2^ test was used to test the association between two categorical variables.

* Statistically significant.
