# Supplementary material for: Circular RNA hsa_circ_101555 promotes hepatocellular carcinoma cell proliferation and migration by sponging miR-145-5p and regulating CDCA3 expression
Source: Cell Death Dis. 2021 Apr 6;12(4):356. doi: 10.1038/s41419-021-03626-7 (PMC8024300; doi:10.1038/s41419-021-03626-7)
Supplement: Supplementary file 10 — Supplement Materials and Methods-Additional file 10 Table S3 [file 41419_2021_3626_MOESM10_ESM.docx]

**Additional file 10: Table S3. Sequence of primers for qRT-PCR.**

| **Gene** | **Forward primer (5’-3’)** | **Reverse primer(5’-3’)** | |
| --- | --- | --- | --- |
| circ101555-P1 (28-317nt) | CTATCAACACGCATCTTGGCAAA | AAAGAGGTCTGTGAAGAGGGTCC | |
| circ101555-P2 (276-600 nt) | CCTGATTATGAGTATTTACGGACCC | AAAGTAAATGTTCGGTCACAGAGG | |
| circ101555-P3 (553-863nt) | CCCTAGCTTGGAGGACTTGTTT | TGCCAAGATGCGTGTTGATAGA | |
| linear 101555 | TGGCAAGGACTCAAGGCTG | CCCAACCCAATCATAGGCATAG | |
| CDCA3 | TGGTATTGCACGGACACCTA | TGTTTCACCAGTGGGCTTG | |
| GAPDH | GGAGCGAGATCCCTCCAAAAT | GGCTGTTGTCATACTTCTCATGG | |
| U6  hsa-miR-145-5p  EIF4A3 | CGCTTCGGCAGCACATATAC CTCACGGTCCAGTTTTCCCA  CGCGGACTCTGACATATGGCGACCACGGCCACGATG | TTCACGAATTTGCGTGTCATC ACCTCAAGAACAGTATTTCCAGG  TCCCGCAGGCCCATGGTGTCG | |
| **Abbreviations:** qRT-PCR, quantitative real-time polymerase chain reaction. | | |  |
