# Supplementary material for: Circular RNA hsa_circ_101555 promotes hepatocellular carcinoma cell proliferation and migration by sponging miR-145-5p and regulating CDCA3 expression
Source: Cell Death Dis. 2021 Apr 6;12(4):356. doi: 10.1038/s41419-021-03626-7 (PMC8024300; doi:10.1038/s41419-021-03626-7)
Supplement: Supplementary file 11 — Supplement Materials and Methods-Additional file 11 Table S4 [file 41419_2021_3626_MOESM11_ESM.docx]

**Additional file 11: Table S4. Antibody for western blotting and immunohistochemistry.**

| **Antibody Dilution** | **Company** | **Cat No.** |
| --- | --- | --- |
| CDCA3 1:300 | Preteintech | 15594-1-AP |
| GAPDH 1:1000 | BOSTER | BM3876 |
| Ki-67 1:200  EIF4A3 1:1000 | BOSTER  Abcam | PB0065  Ab236131 |
| HRP-labeled Goat Anti-Rabbit IgG(H+L) | Beyotime | A0208 |
| HRP-labeled Goat Anti-mouse IgG(H+L) | Beyotime | A0216 |
