# Supplementary material for: Circular RNA hsa_circ_101555 promotes hepatocellular carcinoma cell proliferation and migration by sponging miR-145-5p and regulating CDCA3 expression
Source: Cell Death Dis. 2021 Apr 6;12(4):356. doi: 10.1038/s41419-021-03626-7 (PMC8024300; doi:10.1038/s41419-021-03626-7)
Supplement: Supplementary file 12 — Supplement Materials and Methods-Additional file 12 Table S5 [file 41419_2021_3626_MOESM12_ESM.docx]

**Additional file12: Table S5. Target sequences of siRNA.**

| **siRNA** | **Target sequence** |
| --- | --- |
| si-hsa_circ_101555_001 | GCCTCTTCGAAATCAGGTGAA |
| si-hsa_circ_101555_002 | TTCGAAATCAGGTGAAGGTCT |
| si-hsa_circ_101555_003 | TCTTCGAAATCAGGTGAAGGT |
| MircoRNA inhibitor N.C. | CAGUACUUUUGUGUAGUACAA |
| Hsa-miR-145-5p inhibitor | GUCAAAAGGGUCCUUAGGGA |
| Hsa-CDCA3 -1193 | GCAATTCCTTCCTGGAAT |
|  | GCACCTCTTGTCAGATATA |
| Hsa-CDCA3-1310 | GCAACTGGAGGGTCTTAAA |
|  | GCAGGTGGACTGTTACAAA |
| Hsa-CDCA3-1351 | GCTCTCCTACTCTTGGTAT |
|  | GCTTCCTCATTCGGCTTAT |
| NC | UUCUCCGAACGUGUCACGUTTA |
|  | ACGUGACACGUUCGGAGAATT |
| Hsa-miR-145-5p mimics | GUCCAGUUUUCCCAGGAAUCCCU  CAGGUCAAAAGGGUCCUUAGGGA |
| si-EIF4A3_001 GCAATCCAGCAACGAGCAATC  si-EIF4A3_002 GTGAAACGTGATGAATTGACT  si-EIF4A3_003 GCCATTAACTTTGTAAAGAAT  si-NC GTTCTCCGAACGTGTCACGT | |
