# Supplementary material for: Circular RNA hsa_circ_101555 promotes hepatocellular carcinoma cell proliferation and migration by sponging miR-145-5p and regulating CDCA3 expression
Source: Cell Death Dis. 2021 Apr 6;12(4):356. doi: 10.1038/s41419-021-03626-7 (PMC8024300; doi:10.1038/s41419-021-03626-7)
Supplement: Supplementary file 13 — SUPPLEMENTAL MATERIALSupplementary Figure Legends [file 41419_2021_3626_MOESM13_ESM.docx]

**Supplementary Figure Legends**

**Figure S1. A:** CSNK1G1 was markedly upregulated in HCC tissues compared with normal tissues (***P*<0.01; n=10). **B:** The expression of CSNK1G1 in HCC. **C:** The host gene CSNK1G1 was signiﬁcantly upregulated in HCC tumors was irrelevant with Overall Survival.

**Figure S2. A:** The has_circ_101555 was high expression in HCC tumors was relevant with Overall Survival.

**Figure S3. A：**The expression of hsa_circ_101555 in HCC and health control serum was detected by real-time PCR (****P*<0.001; n=36). **B:** The comparison between the expression level of hsa_circ_101555 in patients’ serum (****P*<0.001; I–II：n=22; III–IV: n=14).

**Figure S4.** **A:** The expression of PCNA in HCCLM3 and HepG2 cells transfected with siRNA-hsa_circ_101555, or siRNA-NC was evaluated by western-blot (****P*<0.001; n=4). **B:** The expression of PCNA in Lv- hsa_circ_101555 transfected Huh-7 and SK-Hep-1 cells was detected by western-blot (****P*<0.001; n=4).

**Figure S5 A-B:** The expression of migration marker (Fibronectin、E-cadherin and Vinmetin) in HCCLM3 and HepG2 cells transfected with siRNA-hsa_circ_101555, or siRNA-NC was evaluated by western-blot (***P*<0.01; ****P*<0.001; n=4). **C-D:** The expression of migration marker (Fibronectin、E-cadherin and Vinmetin) in Lv- hsa_circ_101555 transfected Huh-7 and SK-Hep-1 cells was detected by western-blot (***P*<0.01; ****P*<0.001; n=4).

**Figure S6** **A:** HCCLM3 and HepG2 cells were transfected with control or siRNA targeted eIF4A3 and CSNK1G1 expression was detected by qRT-PCR (***P*<0.01; n=4).. **B:** HCCLM3 and HepG2 cells were transfected with control or eIF4A3 overexpression plasmid, and CSNK1G1 expression was measured by qRT-PCR (***P*<0.01; n=4)..

**Figure S7 A:** HCCLM3 and HepG2 cells were transfected with control or siRNA targeted eIF4A3 and CDCA3 expression was detected by qRT-PCR (****P*<0.001; n=4). **B:** HCCLM3 and HepG2 cells were transfected with control or eIF4A3 overexpression plasmid, and CDCA3 expression was measured by qRT-PCR (****P*<0.001; n=4). **C:** The expression of PCNA in HCCLM3 and HepG2 cells co-transfected with siRNA-hsa_circ_101555 and miR-145-5p inhibitor were evaluated by western-blot (***P*<0.01; ****P*<0.001; n=4).

**Figure S8 A:** Pearson's correlation analyses showing the correlation of hsa-circ-101555 and miR-145-5p expression (n=7). **B:** Pearson's correlation analyses showing the correlation of miR-145-5p and CDCA3 expression (n=7). **C:** Pearson's correlation analyses showing the correlation between hsa-circ-101555 and CDCA3 expression (n=7).

**Figure S9 A:** The expression levels of CDCA3 in multiple HCC cell lines (****P*<0.001; n=6).
