# Supplementary figures and images for: Circular RNA hsa_circ_101555 promotes hepatocellular carcinoma cell proliferation and migration by sponging miR-145-5p and regulating CDCA3 expression
Source: Cell Death Dis. 2021 Apr 6;12(4):356. doi: 10.1038/s41419-021-03626-7 (PMC8024300; doi:10.1038/s41419-021-03626-7)

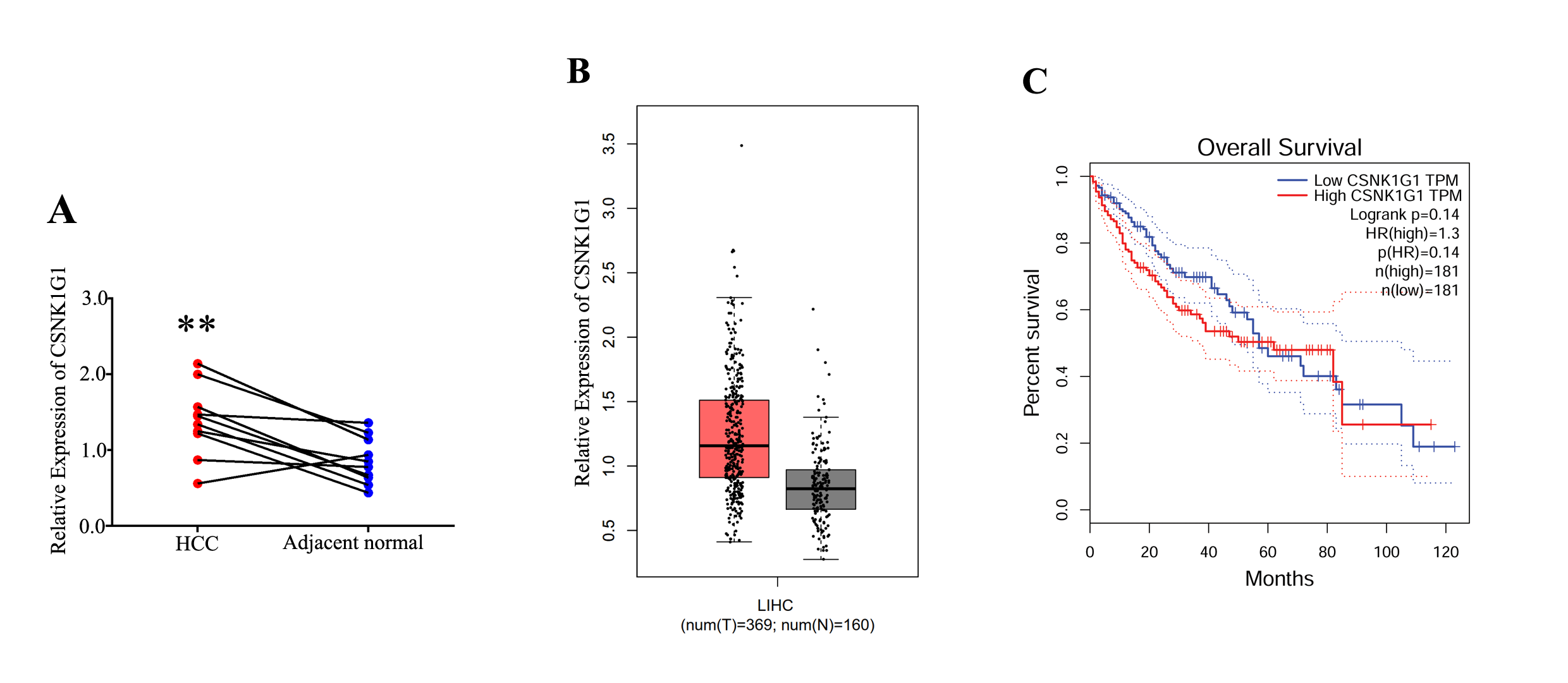

Supplement: Supplementary file 14 — Supplemental Figure 1 [file 41419_2021_3626_MOESM14_ESM.tif]

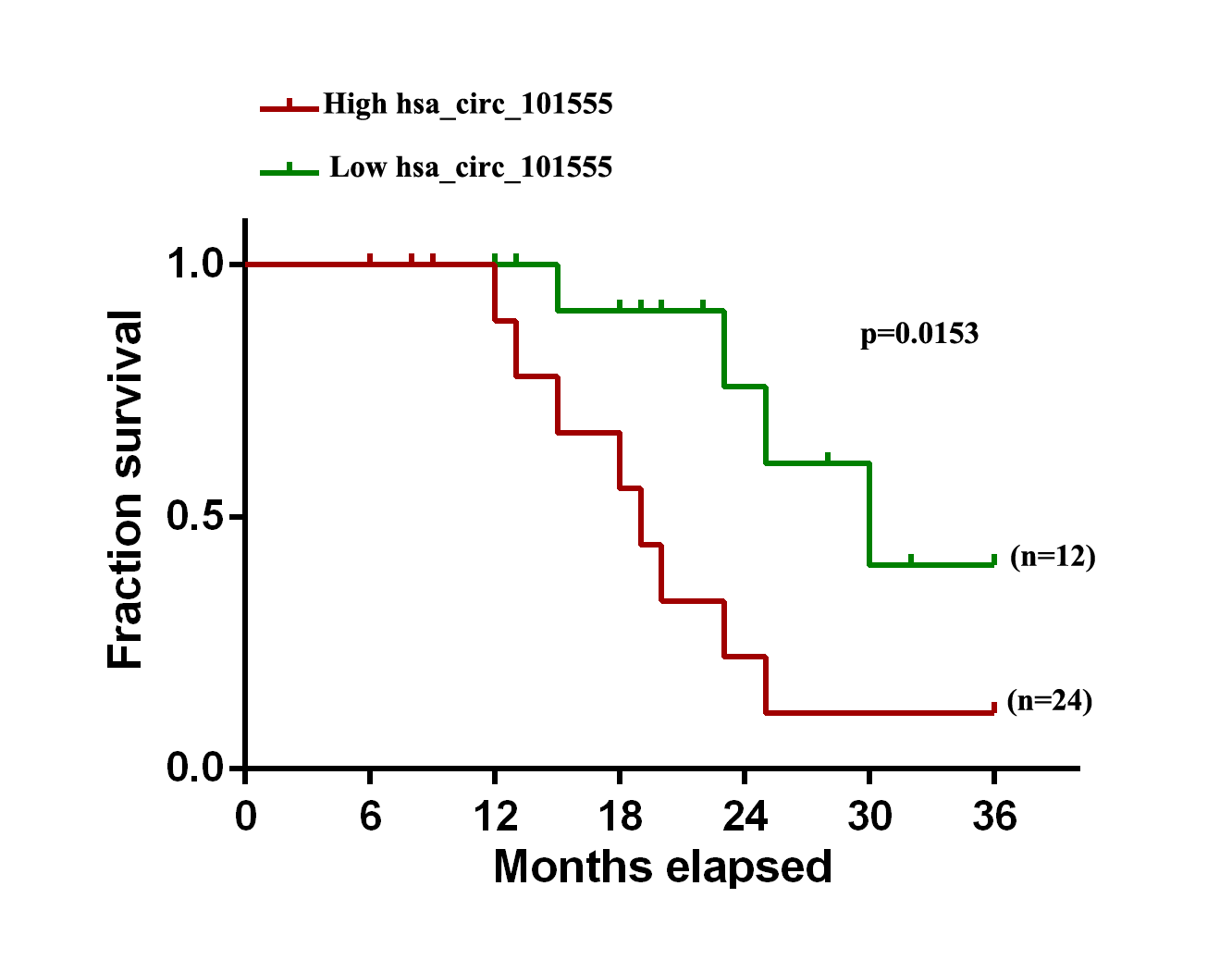

Supplement: Supplementary file 15 — Supplemental Figure 2 [file 41419_2021_3626_MOESM15_ESM.tif]

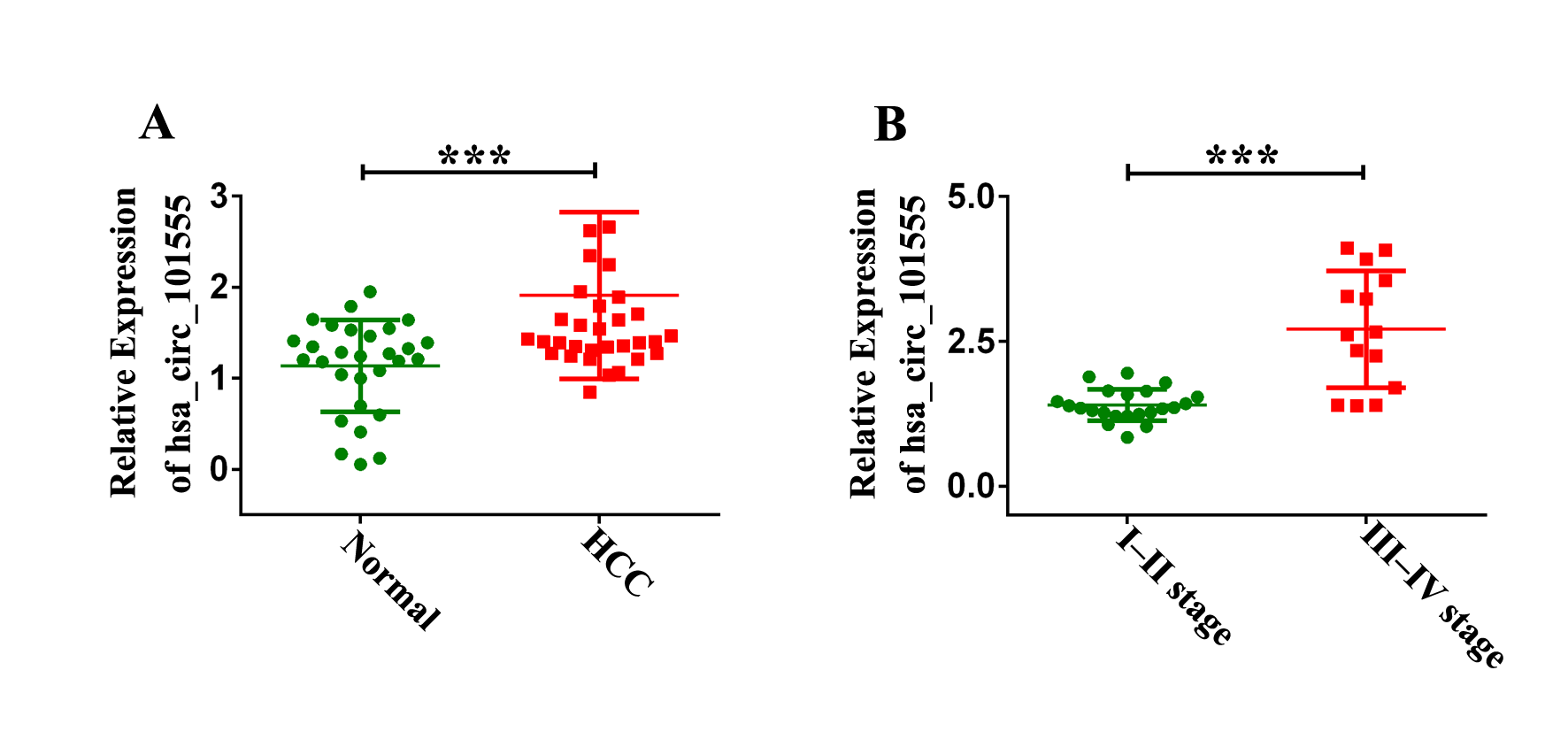

Supplement: Supplementary file 16 — Supplemental Figure 3 [file 41419_2021_3626_MOESM16_ESM.tif]

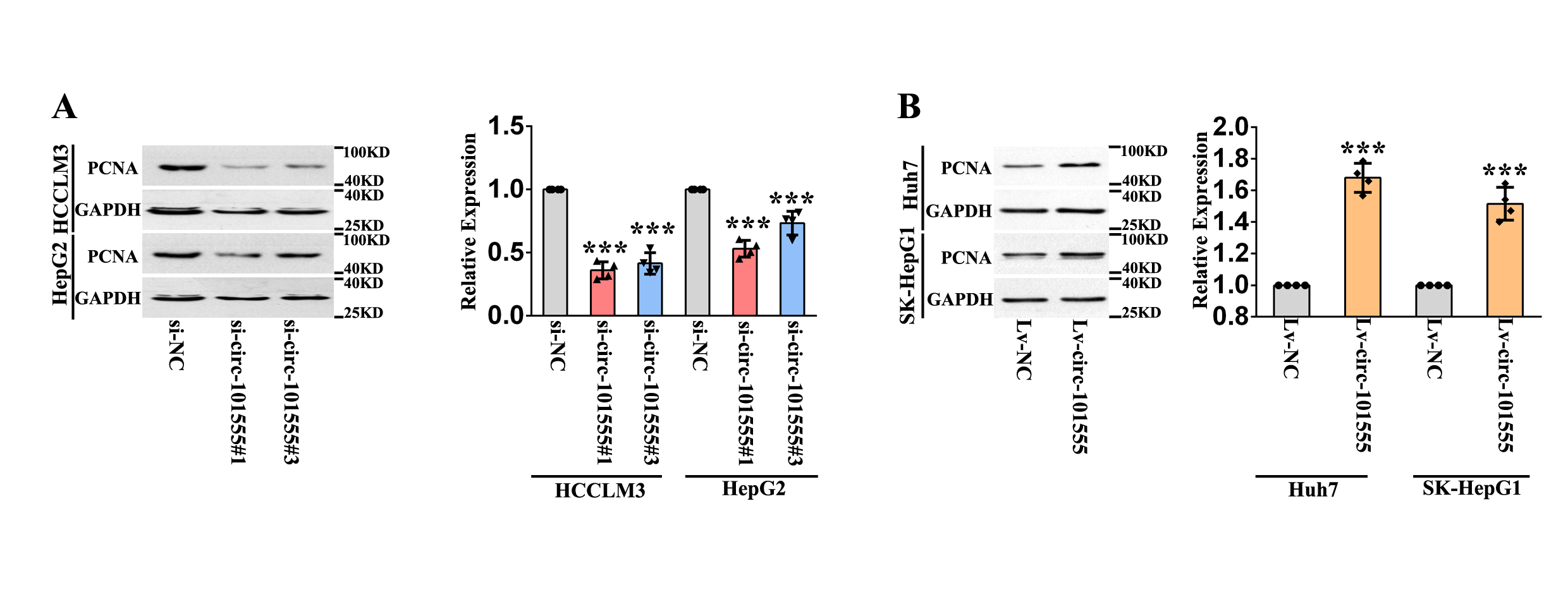

Supplement: Supplementary file 17 — Supplemental Figure 4 [file 41419_2021_3626_MOESM17_ESM.tif]

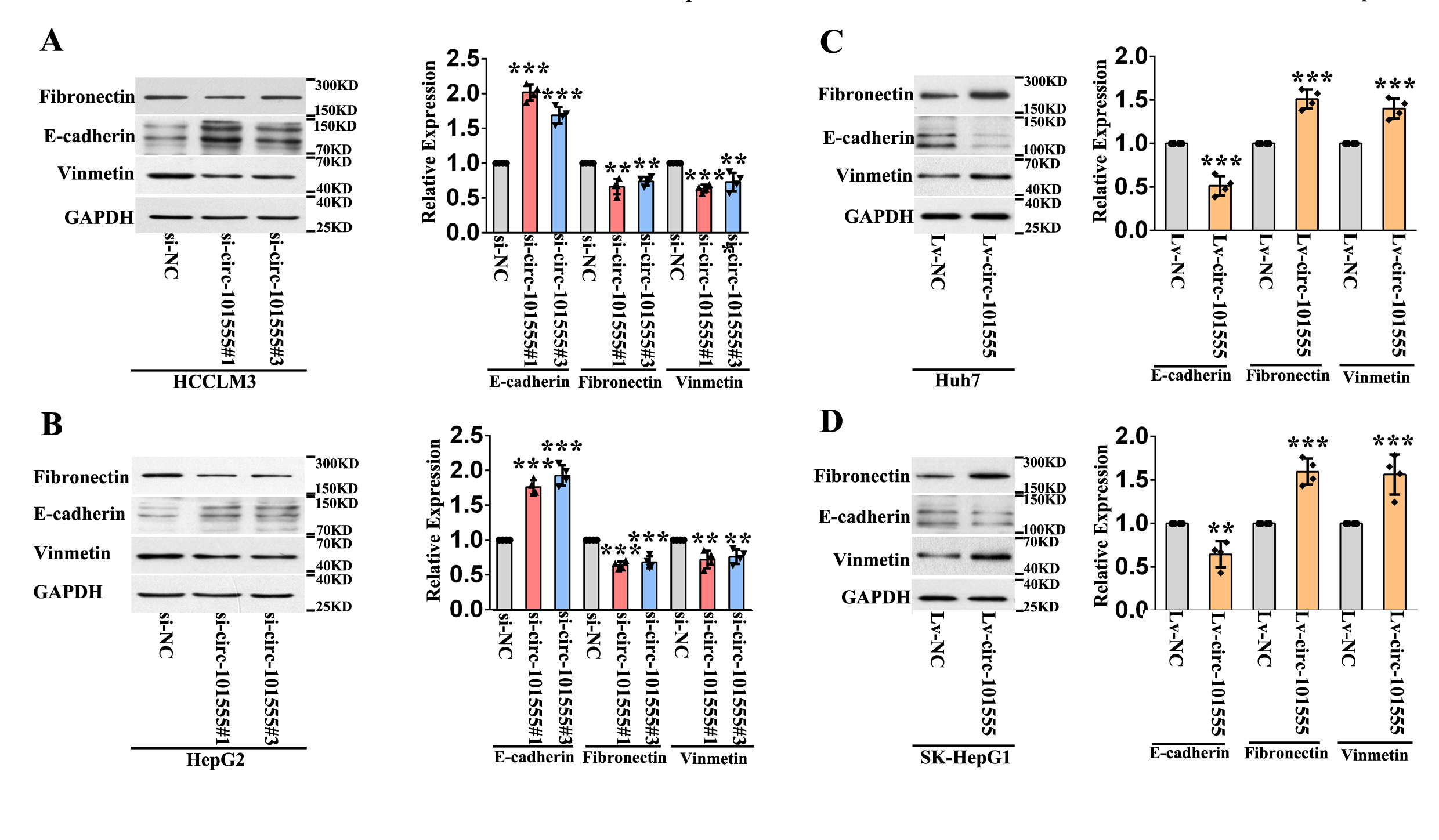

Supplement: Supplementary file 18 — Supplemental Figure 5 [file 41419_2021_3626_MOESM18_ESM.tif]

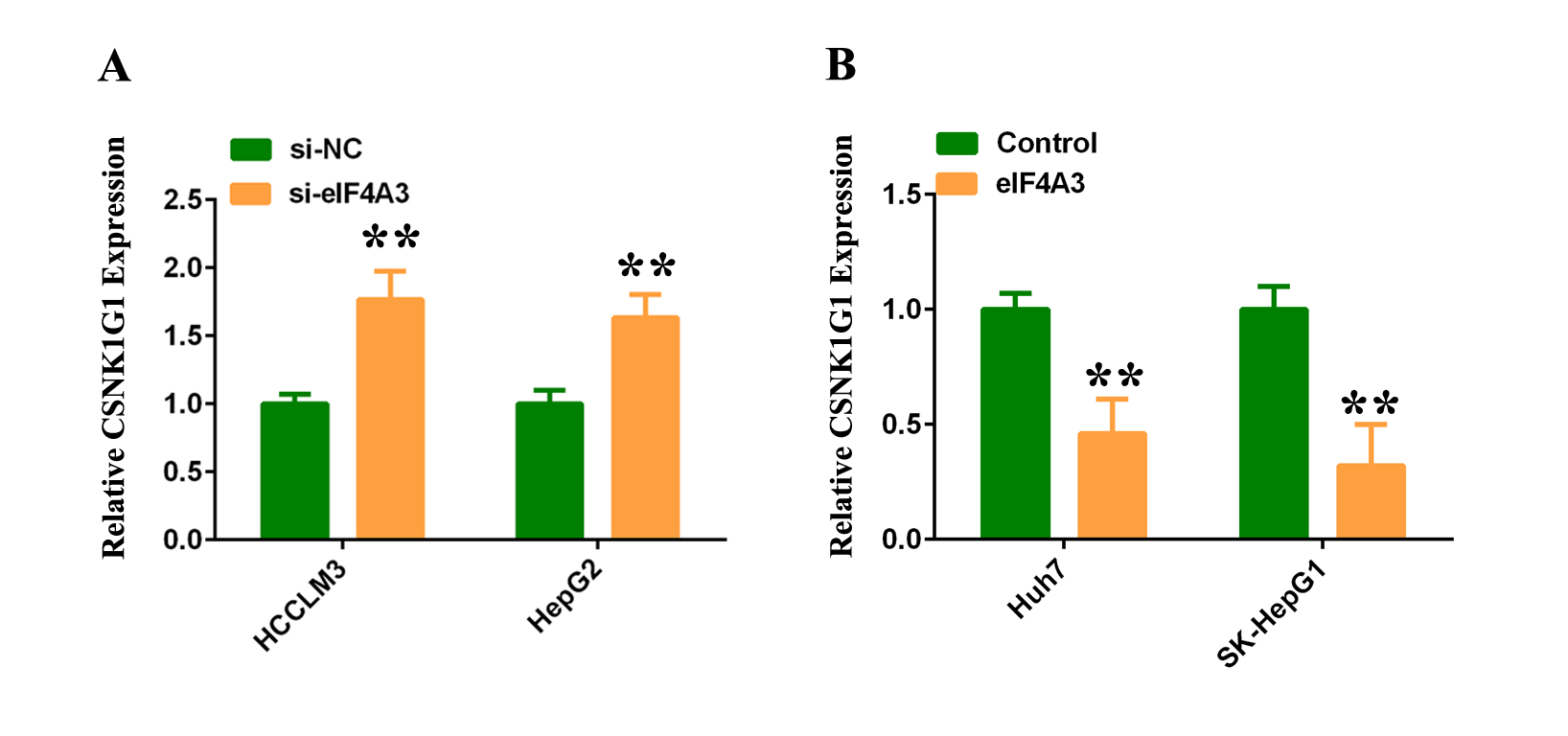

Supplement: Supplementary file 19 — Supplemental Figure 6 [file 41419_2021_3626_MOESM19_ESM.tif]

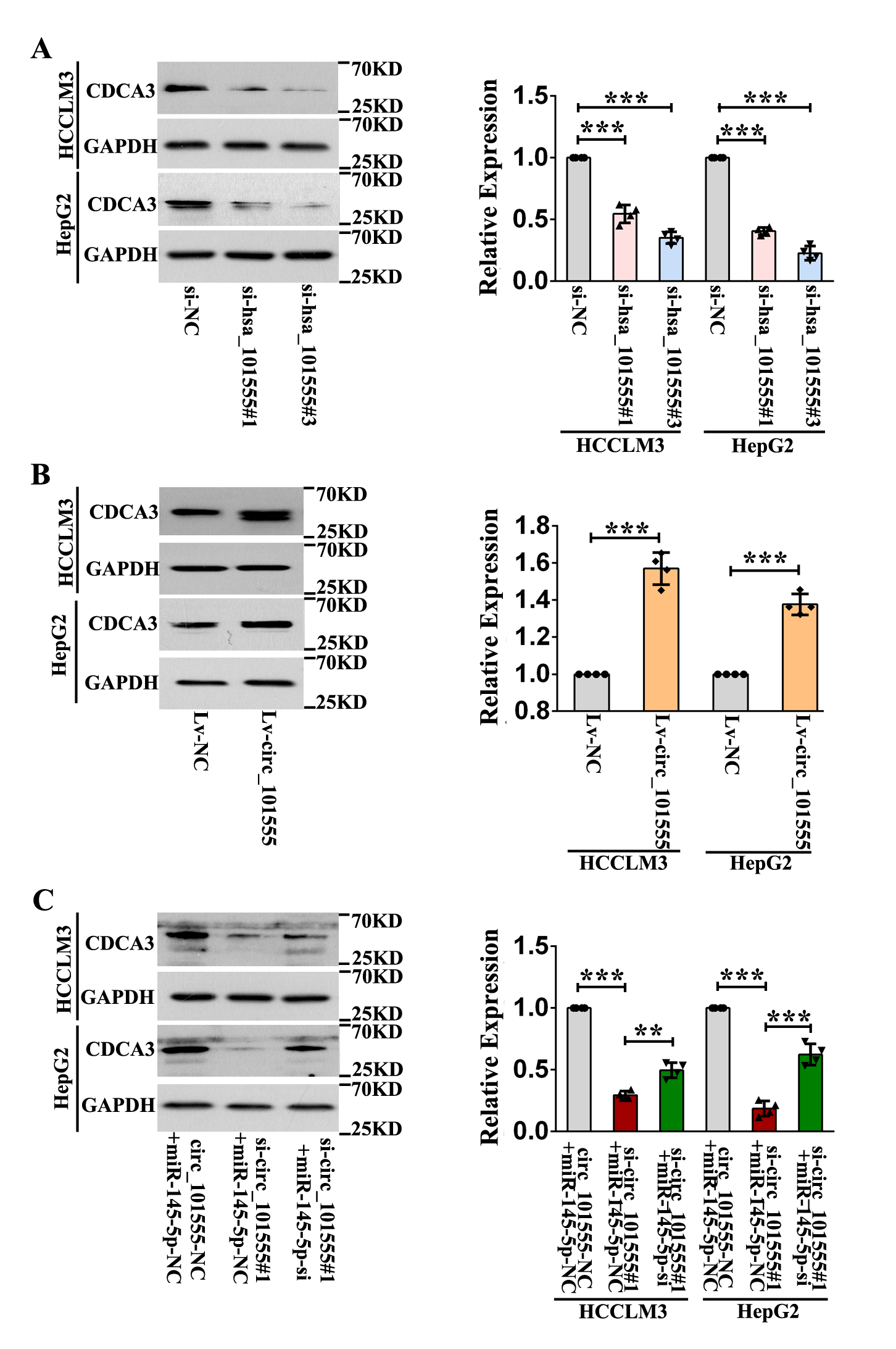

Supplement: Supplementary file 20 — Supplemental Figure 7 [file 41419_2021_3626_MOESM20_ESM.tif]

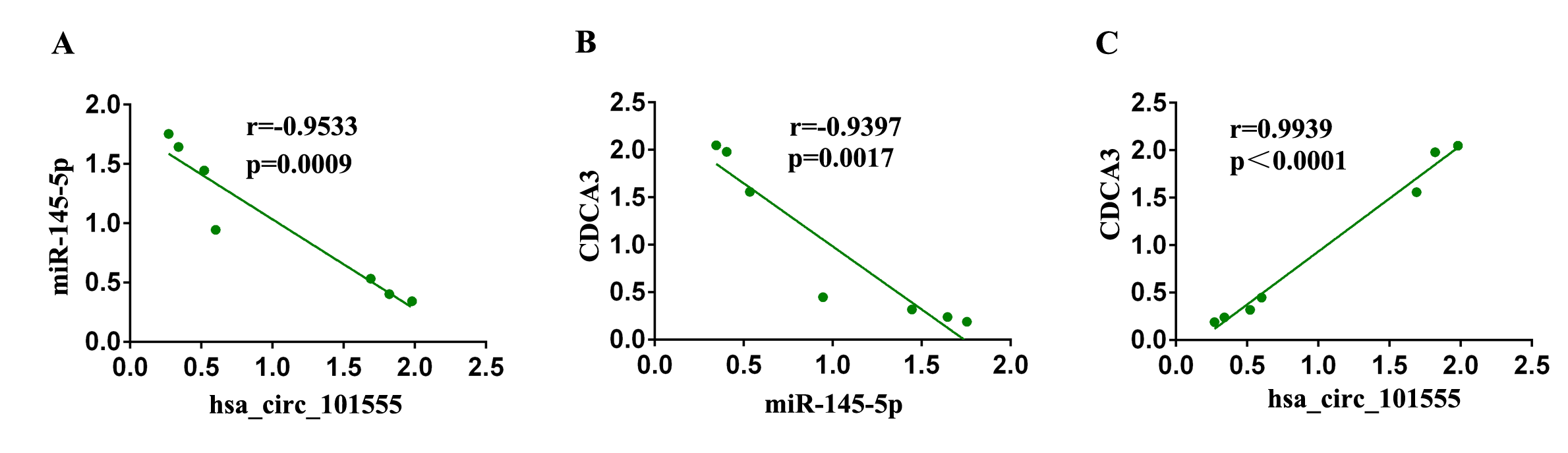

Supplement: Supplementary file 21 — Supplemental Figure 8 [file 41419_2021_3626_MOESM21_ESM.tif]

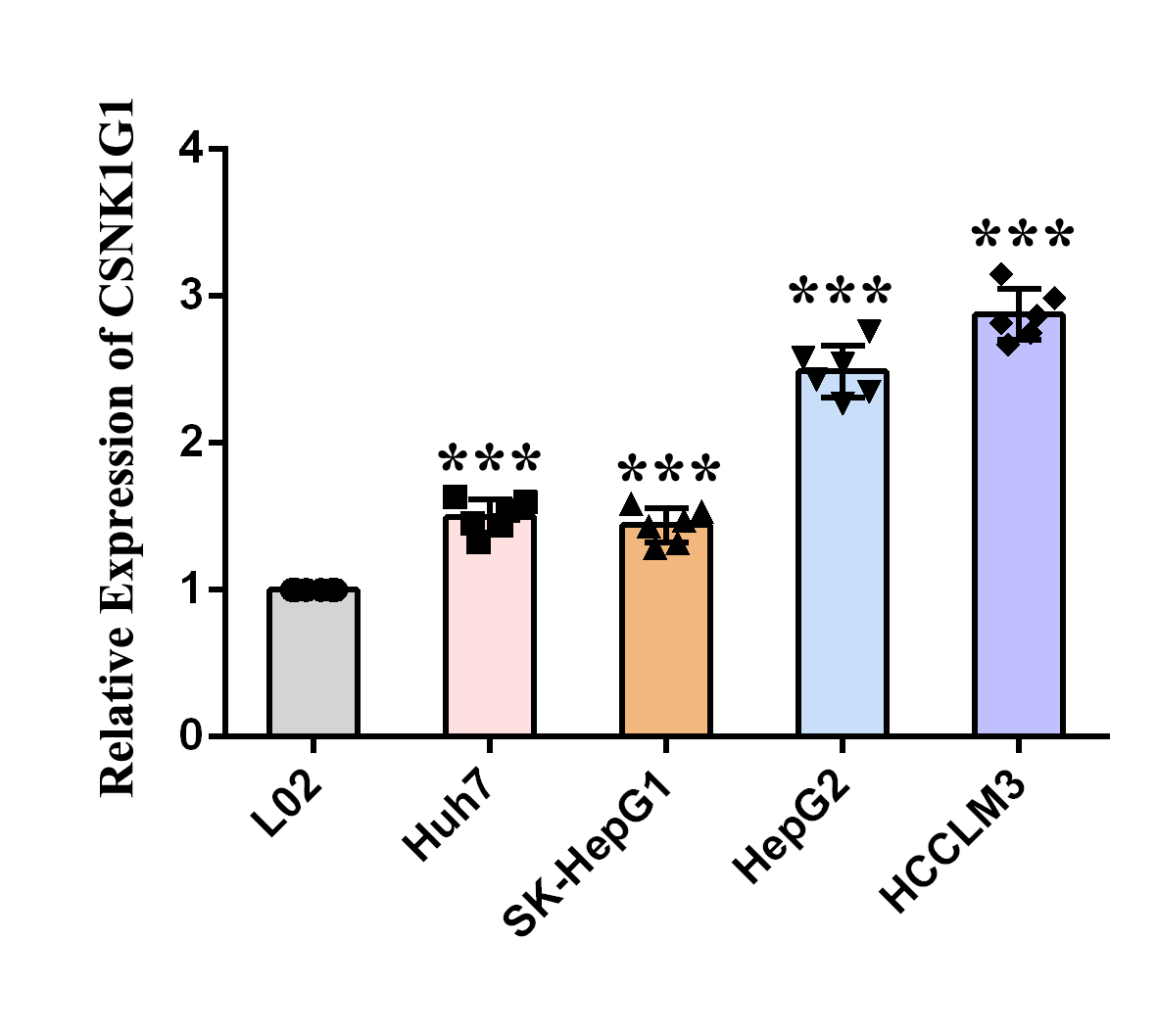

Supplement: Supplementary file 22 — Supplemental Figure 9 [file 41419_2021_3626_MOESM22_ESM.tif]
